# Unveiling the glymphatic system’s role in brain aging: A comprehensive biomarker and modifiable intervention target

**DOI:** 10.1073/pnas.2516601123

**Published:** 2026-04-27

**Authors:** Yuanyuan Fang, Wenxi Luo, Hao Huang, Lusen Ran, Yuqin He, Chang Cheng, Yao Yao, Yuxiang Hou, Haibo Zheng, Dengji Pan, Shabei Xu, Xiang Luo, Tingting Qin, Xingjie Hao, Feng Lu, Wei Wang, Minghuan Wang

**Affiliations:** ^a^Department of Neurology, Tongji Hospital, Tongji Medical College, Huazhong University of Science and Technology, Wuhan 430030, China; ^b^Hubei Key Laboratory of Neural Injury and Functional Reconstruction, Huazhong University of Science and Technology, Wuhan 430030, China; ^c^College of Life Science and Technology, Huazhong University of Science and Technology, Wuhan 430030, China; ^d^School of Computer Science and Technology, National Engineering Research Center for Big Data Technology and System, Services Computing Technology and System Lab, Cluster and Grid Computing Lab, Huazhong University of Science and Technology, Wuhan 430030, China; ^e^Clinical Research Center, Tongji Hospital, Tongji Medical College, Huazhong University of Science and Technology, Wuhan 430030, China; ^f^Department of Epidemiology and Biostatistics, Ministry of Education Key Laboratory of Environment and Health, School of Public Health, Tongji Medical College, Huazhong University of Science and Technology, Wuhan 430030, China

**Keywords:** brain age gap, glymphatic system, DTI-ALPS index, aggressive blood control, sex-stratified differences

## Abstract

The focus of this study is to investigate the role of diffusion tensor imaging along the perivascular space (DTI-ALPS) index in brain aging. To address this, we first examined the association of DTI-ALPS with aging hallmarks among 40,488 UK Biobank (UKB) participants. Next, we developed normative brain age models incorporating the DTI-ALPS index from 12,401 healthy UKB adults and validated in UKB-ADNI and UKB-TALENT datasets. Finally, we explored the relationship between brain age gap (BAG) with peripheral organ function, chronic diseases, proteomics, and genetics, while identifying modifiable factors in a longitudinal cohort. The findings revealed that DTI-ALPS index correlated with chronological age, telomere length, brain structure, and cognition. A brain age model integrating the DTI-ALPS index achieved good accuracy in the UKB (*r* = 0.756) and replicated well in two independent datasets (UKB-ADNI: *r* = 0.766; UKB-TALENT: *r* = 0.724), with choroid plexus volume emerging as an additional contributor. Musculoskeletal health was a key driver for brain aging in females, while pulmonary metrics prevailed in males. Neurodegenerative and metabolic disorders increased BAGs, imparting increased mortality risk. Protein-wide and genome-wide analysis identified 154 BAG-related proteins and 11 loci. Modifiable factors, particularly systolic blood pressure below 120 mmHg, were strongly associated with reduced BAGs. Overall, the DTI-ALPS index is a promising brain aging biomarker, offering insights into links between brain and peripheral health, and highlighting sex-stratified therapeutic strategies. Aggressive blood pressure control may mitigate brain aging and promote long-term brain health.

## Significance

Despite efforts to develop brain aging biomarkers, a critical gap remains in establishing models that are biologically interpretable, therapeutically actionable, and broadly generalizable. Herein, we address this challenge by identifying diffusion tensor imaging along the perivascular space (DTI-ALPS) index and choroid plexus volume as novel biomarkers, linking it to cognitive decline, systemic health deterioration, and higher mortality. Integrating DTI-ALPS index into a multimodal brain age framework, we achieved good predictive accuracy across three independent cohorts and uncovered sex-specific aging drivers. Importantly, we demonstrated that maintaining systolic blood pressure below 120 mmHg was linked to a 1.5 y reduction in brain age. These findings advance both mechanistic understanding and clinical translation, offering direct intervention targets for preserving brain health.

Brain aging manifests as morphological changes such as cortical atrophy, pathological accumulation of abnormal proteins, and altered physiological functions such as perfusion, metabolism, and neurotransmission (1, 2). As distinct from healthy brain aging, pathological brain aging contributes to a high prevalence of disability and premature death among the elderly (3, 4). Timely detection and proactive management of brain aging could thus provide a channel for the prevention of neurodegenerative disorders and mortality in the elderly (5). Decades of neuroimaging research inspired the concept of brain age (or rather the brain age gap (BAG), as distinct from calendar age), which derived from multimodal MRI, combined with machine learning models (6–8). Meanwhile, advances in proteomics technologies have provided molecular insights into the time course of brain aging, and have uncovered specific plasma protein markers linked to peaks in brain aging rate (5). To extend these efforts, improved models should be able to characterize the population heterogeneity of brain aging and provide a more comprehensive and actionable platform for understanding and mitigating age-related brain decline through individualized lifestyle interventions.

Dysfunction of the glymphatic system (GS) is associated with brain aging and various neurodegenerative diseases (9, 10). Indeed, a progressive reduction in glymphatic function was observed in the aging human brain (11). Additionally, genetics, certain diseases, and potentially modifiable risk factors and lifestyle choices may influence GS function (12, 13). For example, reduced arterial pulsations due to loss of vessel elasticity can slow glymphatic flow, while hypertension and poor sleep quality can independently impair glymphatic transport (14, 15). However, the direct approach to detect glymphatic function through the intrathecal administration of gadolinium-based contrast agent is an unapproved and generally unavailable practice, which restricts the comprehensive evaluation of the GS in the human brain. The diffusion tensor image analysis along the perivascular space (DTI-ALPS) index is a noninvasive diffusion-based representation of the integrity of the glymphatic pathway (16). It is calculated from the diffusivity along the deep medullary vein at the level of the lateral ventricular body, and correlates well with direct imaging techniques involving intrathecally administered contrast agents (17). Nevertheless, a recent study by Ringstad et al. reported a limited association between the ALPS index and intrathecal contrast-enhanced MRI markers of glymphatic function in patients with cerebrospinal fluid disorders (18), indicating that the efficacy of the ALPS index for measuring human glymphatic function remains to be fully and rigorously verified (19, 20). Even without invoking its relationship to glymphatic function, the ALPS index holds sufficient merit for inclusion in brain age models due to its associations with neurodegeneration (21). Particularly, a preliminary study demonstrated that treatment for moderate obstructive sleep apnea led to a significant improvement in the DTI-ALPS index and cognitive performance (22). In addition, the choroid plexus (ChP), as an important site of cerebrospinal fluid (CSF) production, is critical for peripheral-central immune surveillance and the integrity of the blood–CSF barrier (23–25). As such, ChP enlargement has been linked to disturbances in cerebral fluid dynamics and impaired GS function, further exacerbating white matter hyperintensities (26).These basic results present DTI-ALPS as a straightforward and representative brain aging biomarker that is clinically interpretable, and amenable to intervention.

**Fig. 1.**
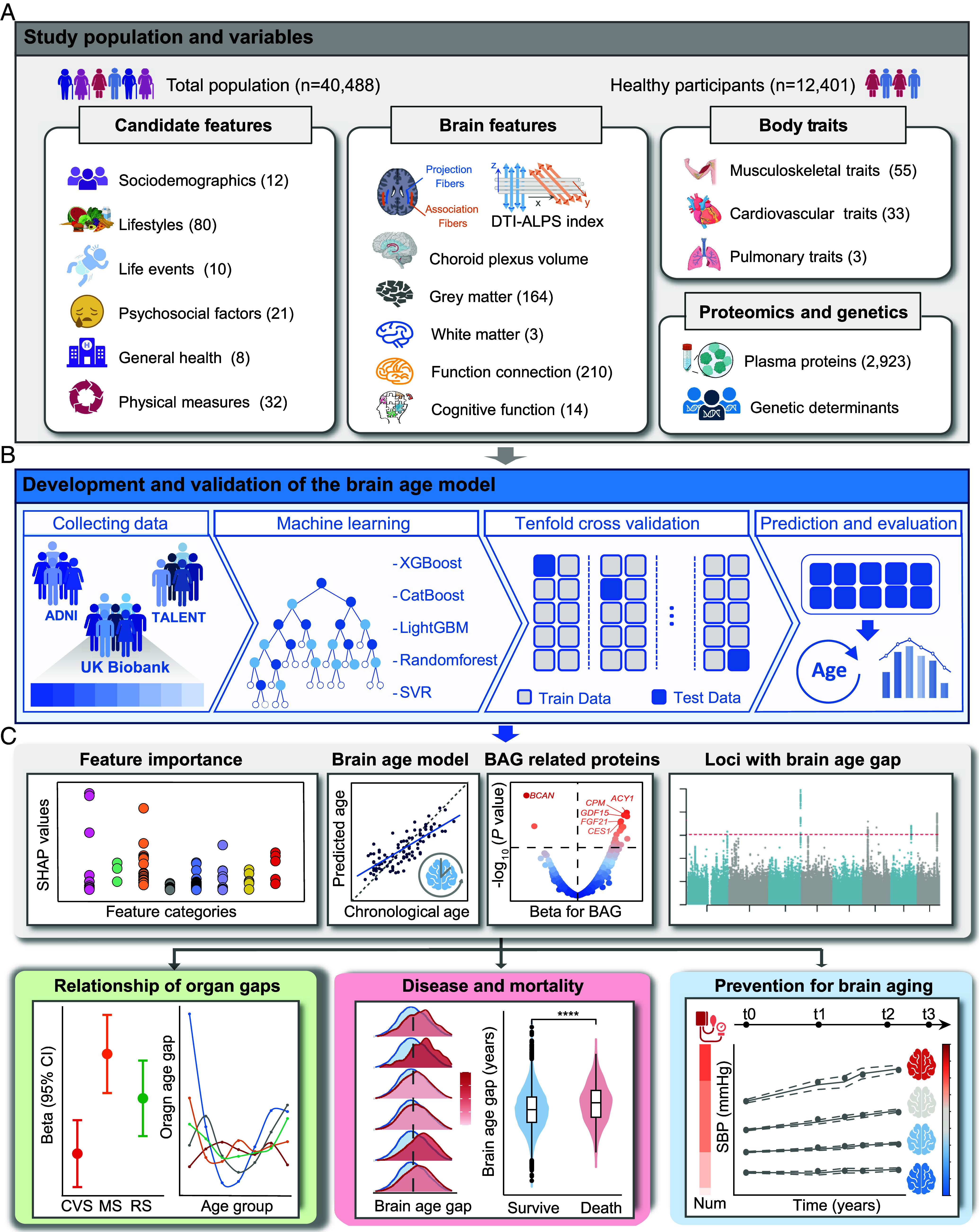
Graphical abstract of the study. (*A*) The *Top* panel illustrates the study populations and variables. (*B*) The middle panel outlines the development and validation of brain age model from three independent cohorts using five distinct machine learning algorithms, along with 10-fold cross-validation for model evaluation. (*C*) The *Bottom* panel highlights the main research contents. First, we identify the important features of DTI-ALPS index. Next, we establish a brain age model incorporating DTI-ALPS and provide an index namely BAG to assess brain aging. Then, we conduct protein-wide and genome-wide association analysis of BAG. Finally, we investigate the relationship of brain age and peripheral organ aging, chronic diseases, mortality risk, and modifiable factors. DTI-ALPS, diffusion tensor imaging along perivascular spaces; ADNI, Alzheimer’s Disease Neuroimaging Initiative; TALENT, Tongji cerebrAl smalL vEssel disease and agiNg cohort; XGBoost, eXtreme Gradient Boosting; CatBoost, Categorical Boosting; LightGBM, Light Gradient Boosting Machine; SVR, Support Vector Regression; SHAP, SHapley Additive exPlanations; BAG, Brain age gap; CVS, Cardiovascular system; MS, Musculoskeletal system; RS, Respiratory system.

In this study, we leveraged five machine learning algorithms to identify key determinants of the DTI-ALPS index and their links to aging hallmarks within the UK Biobank (UKB) cohort. Following its validation as a physiologically meaningful measure, we developed a brain age model based on the DTI-ALPS index or ChP volume, with validation in two independent cohorts. Building upon this model, we systematically elucidated the interplay between peripheral organ health and brain aging processes through integration of phenotypic, proteomic, and genomic analyses. Finally, we identified modifiable factors and their longitudinal trajectories, placing particular focus on potential targeted interventions to mitigate brain aging and associated diseases (Fig. 1).

## Results

### Characteristic of the Study Population.

This study utilized data from the UKB across multiple analytic phases. The DTI-ALPS index prediction model was developed using 40,488 participants (mean age ± SD: 64.02 ± 7.68 y; 46.99% males). Of the 40,374 participants with multimodal brain imaging, 12,401 healthy individuals (61.59 ± 7.38 y; 45.35% males) were included to predict a normative brain age, while the remaining 27,973 participants with medical conditions served as the clinical validation set. Cognitive and body systems phenotypes were available for 11,271 and 11,690 to 13,214 healthy individuals, respectively. After excluding cases with incomplete multimodal brain imaging or plasma proteomics data, there remained 5,302 individuals (63.19 ± 7.91 y, 46.53% males) for testing protein-wide associations of BAGs. Genome-wide analysis of BAGs involved 31,612 European individuals (64.27 ± 7.58 y, 47.84% males), and longitudinal assessment included 1,364 participants (59.73 ± 6.97 y; 44.50% males) with follow-up data. Detailed sample selection is provided in *SI Appendix*, Fig. S1 and Dataset S1. The Alzheimer’s Disease Neuroimaging Initiative (ADNI) dataset comprised 714 participants (mean ± SD, 73.81 ± 7.73; 340 males), consisting of 372 healthy controls, 252 with mild cognitive impairment (MCI), and 90 Alzheimer’s disease (AD) cases. The Tongji cerebrAl smalL vEssel disease and agiNg cohorT (TALENT) study enrolled 275 participants (mean ± SD, 66.89 ± 6.14; 164 males), with 204 healthy controls and 71 MCI cases (*SI Appendix*, Fig. S2 and Dataset S2).

### DTI-ALPS as an Efficient Candidate Marker for Brain Aging.

**Fig. 2.**
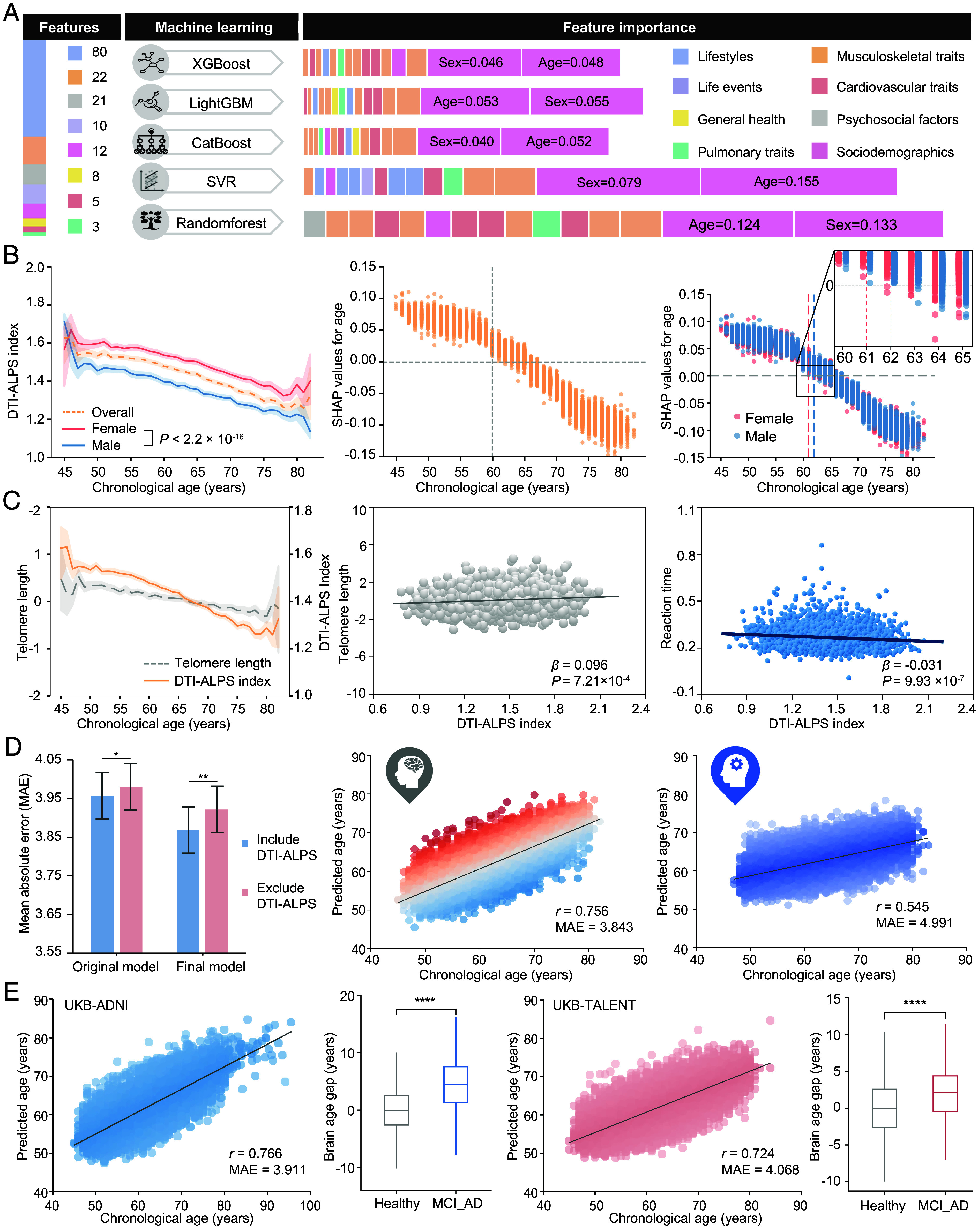
DTI-ALPS as an efficient candidate marker for brain aging. (*A*) The top 15 features are sorted by their feature importance to five machine learning models for DTI-ALPS index prediction task. (*B*) The *Left* panel presents the declining DTI-ALPS index with increasing age in the total population, and separately for the male and female populations. Significant associations determined by the *t* test. The *Middle* and *Right* panels reveal total and sex-stratified SHAP values of the feature “chronological age” for predicting DTI-ALPS. Sex-specific SHAP value transition points occur at approximately 61 y for females and 62 y for males. (*C*) Line chart illustrates associations of telomere length and DTI-ALPS index with chronological age. The scatter plots demonstrate associations of telomere length and reaction time with DTI-ALPS index. Chronological age, sex, TDI, and ethnicity are adjusted, with education further adjusting for reaction time. (*D*) Bar plots illustrate the comparative model performance of the brain age prediction with and without the DTI-ALPS feature, evaluated using the MAE. The “Original model” refers to the brain age model incorporating 378 multimodal neuroimaging features along with four demographic variables. The “Final model” denotes a refined version that retained 111 selected features. Statistical significance determined by the Wilcoxon signed-rank test is denoted by asterisks (*P < 0.05, **P < 0.01). Scatter plots show correlation between chronological and predicted age for prediction models based on brain (Mid) and cognitive (*Right*) phenotypes. Lines of best fit are indicated with solid black lines. *r*, Pearson correlation coefficient; MAE, mean absolute error. (*E*) Prediction of brain age in the external validation dataset (the ADNI and TALENT study) using multimodal brain IDPs. *r* and MAE between predicted brain age and chronological age are shown in the plot. The box plots display the difference in brain age gaps between healthy individuals and those diagnosed with MCI and dementia. Significant associations determined by the *t* test are marked by asterisks (*P < 0.0001).

The DTI-ALPS index, as predicted using 161 features by five machine learning models (Datasets S3 and S4), showed age and sex as the top two predictors (Fig. 2*A* and *SI Appendix*, Fig. S3), aligning with linear regression model (Dataset S5). To be specific, the DTI-ALPS index declined with age, but was consistently higher in females than males (*P* < 2.2 × 10^−16^). Notably, both sexes demonstrated accelerated decline after 60 y, with women’s transition (at 61 y) occurring approximately 1 y earlier than for men (at 62 y) (Fig. 2*B*). DTI-ALPS correlated positively with telomere length (*β* = 0.096, *P* = 7.21 × 10^−4^) (Fig. 2*C*), which act as a “biological clock” for aging process (27). Additionally, the DTI-ALPS was associated with 265 out of 378 brain imaging-derived phenotypes (IDPs) (*β* range −0.403 to 0.233, *P* < 1.32 × 10^−4^) (Dataset S6), and lower DTI-ALPS index was linked to worse cognitive performance (e.g., reaction time, *β* = −0.031, *P* = 9.93 × 10^−7^) (Fig. 2*C* and Dataset S7). These findings position DTI-ALPS as a robust biomarker of brain aging, capturing sex-specific trajectories and neurostructural changes.

Thus, we developed a multimodal original brain age model using 378 imaging features (gray/white matter, functional connectivity, DTI-ALPS) and demographic data, achieving a relatively good accuracy (*r* = 0.753, mean absolute error, MAE = 3.868). Feature selection refined the final model to 111 features (*r* = 0.756, MAE = 3.843). The model incorporating DTI-ALPS yield a significant reduction in MAE (Original model: 3.957 vs. 3.980, *P* = 0.037; Final model: 3.868 vs. 3.921, *P* = 0.008), demonstrating its significant incremental value over the conventional model, with removal of DTI-ALPS (Fig. 2*D*, *SI Appendix*, Figs. S4 and S5*A*, and Datasets S8 and S9). A parallel cognitive age model showed *r* = 0.545 (MAE = 4.991) (Fig. 2*D*). Validation in UKB-ADNI (*r* = 0.766, MAE = 3.191) and UKB-TALENT (*r* = 0.724, MAE = 4.068) cohorts confirmed consistent age-prediction accuracy (Dataset S10). The models revealed significantly advanced BAGs in MCI and AD patients compared to age-matched healthy controls (UKB-ADNI: 4.354 ± 5.020 y, *P* < 2.2 × 10^−16^; UKB-TALENT: 2.204 ± 4.040 y, *P* = 7.3 × 10^−7^) (Fig. 2*E*). Sensitivity analysis incorporating additional vascular risk factors yielded a stable model performance (*r* = 0.772, MAE = 3.737), while affirming the primary conclusion of significantly advanced brain aging in dementia (mean age gap of 2.420 ± 3.854 y, *P* = 0.005) (*SI Appendix*, Fig. S5 *B* and *C*). Additionally, substituting DTI-ALPS with ChP volume in the above model also yielded a comparable-accuracy brain age prediction (*r* = 0.752, MAE = 3.881) (*SI Appendix*, Fig. S5 *D* and *E* and Dataset S9). When using corrected ALPS (cALPS) instead of the original ALPS index, brain age model performance remained consistent (cALPS: *r* = 0.763, MAE = 3.913 y vs. ALPS: *r* = 0.764, MAE = 3.912 y; *SI Appendix*, Fig. S6).

### Specific Organ Age Gap on BAGs Derived from the DTI-ALPS.

The effect of organ-specific features on DTI-ALPS using the eXtreme Gradient Boosting (XGBoost) model were sorted by their feature importance (*SI Appendix*, Fig. S7*A* and Dataset S3). The top 15 predictors of DTI-ALPS were ranked by SHAP value in a bar chart. Except for age, sex, and employment status, musculoskeletal (waist-to-hip ratio, WHR), cardiovascular (systolic blood pressure, SBP), pulmonary traits (peak expiratory flow, PEF), and walking pace also made substantial contributions to predicting DTI-ALPS (*SI Appendix*, Fig. S7*B*). Gender-stratified analyses showed that BMI, WHR, SBP and PEF ranked higher in women, while pulse rate and hand grip strength were more influential in men (*SI Appendix*, Fig. S7 *C* and *D*).

**Fig. 3.**
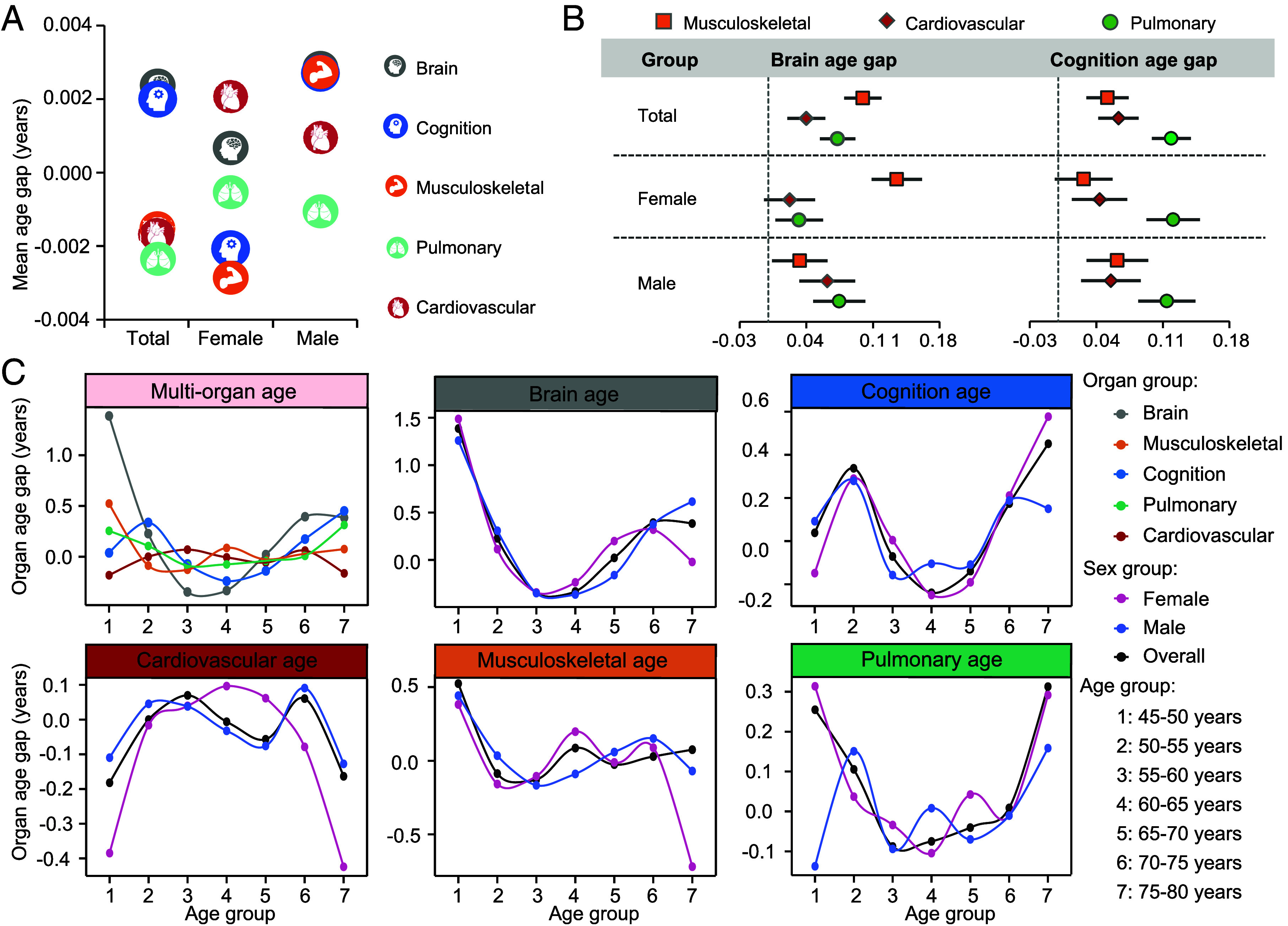
Specific organ age on BAGs derived from the DTI-ALPS. (*A*) The mean age gaps of various organs among participants of different genders. (*B*) Forest plots show Beta (95% CI) of associations of musculoskeletal, cardiovascular, and pulmonary age gaps with brain and cognitive age gaps in different gender groups, controlling for chronological age and sex. (*C*) The fluctuations of multiorgan age gaps among people of different age and gender groups, where the dots represent the average age gaps for the corresponding age groups.

Given the close relationships between musculoskeletal, cardiovascular, and pulmonary traits and the DTI-ALPS index, we developed musculoskeletal (*r* = 0.808, MAE = 3.475), cardiovascular (*r* = 0.646, MAE = 4.596), and pulmonary (*r* = 0.477, MAE = 5.387) age models to examine their links with BAGs (*SI Appendix*, Fig. S8 and Dataset S11). Gender differences were observed, where cardiovascular age appeared oldest in women, but brain and musculoskeletal age appeared oldest in men (Fig. 3*A* and Dataset S12). Musculoskeletal age showed the strongest association with BAG, particularly in women (*β* = 0.135, *P* = 3.44 × 10^−23^), whereas pulmonary age had the strongest impact in men (*β* = 0.075, *P* = 1.07 × 10^−7^) (Fig. 3*B* and Dataset S13).

Furthermore, we uncovered nonlinear aging trajectories across multiple organs, wherein the BAG demonstrated more pronounced fluctuations. BAG peaked around 45 to 50 y, then declined until 60 y, with women showing accelerating BAG post-60 y. Cognitive and musculoskeletal aging followed similar patterns in both sexes. Cardiovascular aging peaked around 60 to 65 y for females and 70 to 75 y for males. Pulmonary aging followed a U-shaped trend in women, but wave-like increase in men. Therefore, aging was nonlinear and sex-specific (Fig. 3*C* and Dataset S12).

### BAGs in Organ-Specific Chronic Disease and Predict Mortality Risk.

**Fig. 4.**
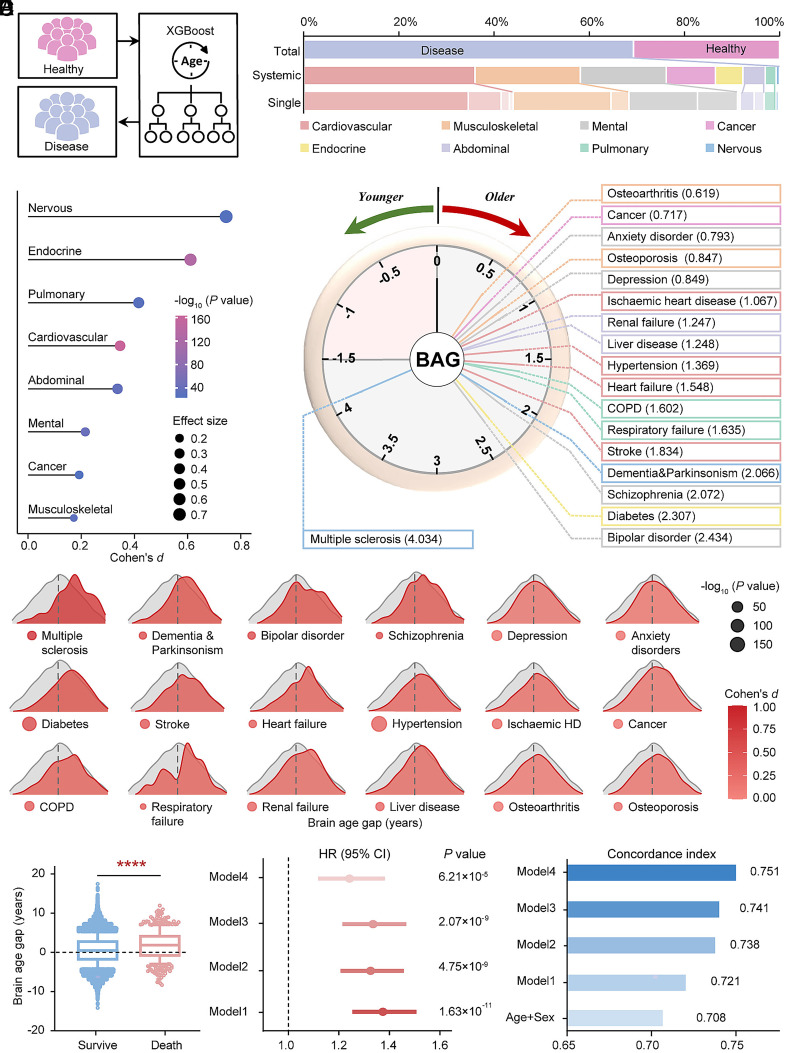
BAGs in organ-specific chronic disease and predict mortality risk. (*A*) Brain age was estimated in 19 chronic diseases covering eight systems using the normative models previously established in healthy individuals by XGBoost model. Stacked bar chart shows the number and percentage of single and systemic diseases. (*B*) The effect size of differences in BAGs between each systemic disease and healthy group is quantified using the Cohen’s *d* (*t* test, Bonferroni corrected *P* < 6.25 × 10^−3^). (*C*) The clock plot shows the distribution of mean BAGs in 19 chronic diseases, with specific values listed. (*D*) The distributions of BAGs are significantly different between the healthy group (gray) and each of the 19 chronic disease groups (*t* test, Bonferroni-corrected *P* *<* 2.78 × 10^−3^). Warmer colors denote larger Cohen’s *d* values. (*E*) BAGs in deceased compared to surviving individuals. Asterisks indicates significant between-group differences (*P* < 0.0001, two-sided, *t* test). Forest plots show HR (95% CI) of association between BAG and mortality risk, and bar plots show its corresponding concordance index. Chronological age and sex are included in the model 1 regression. Model 2 further adjusts DTI, education, and ethnicity; model 3 further adjusts smoke status, alcohol frequency, SBP and BMI; and model 4 further adjusts existing disease diagnoses. BAG, Brain age gap; COPD, Chronic obstructive pulmonary disease; IHD, Ischemic heart disease; MCI, Mild cognitive impairment.

Using healthy normative models, we estimated BAGs for each disease and category (Fig. 4*A* and Dataset S14). On average, diseased individuals had a 0.748-y BAG greater than healthy peers (*SI Appendix*, Fig. S9*A*). Nervous system disorders showed the largest BAG (overall: 3.220 y, Cohen’s *d* = 0.745; dementia and parkinsonism: 2.066 y, Cohen’s *d* = 0.586), followed by bipolar disorder (2.434 y, Cohen’s *d* = 0.590). Interestingly, nonneurological diseases also accelerated aging, including endocrine (diabetes, 2.307 y, Cohen’s *d* = 0.611), pulmonary (specifically respiratory failure, 1.635 y, Cohen’s *d* = 0.410), and cardiovascular disorders (particularly stroke, 1.834 y, Cohen’s *d* = 0.478; heart failure, 1.548 y, Cohen’s *d* = 0.412) (Fig. 4 *B*–*D* and Dataset S15). Multisystem comorbidities further amplified aging effects (2.550 y). For example, individuals with concurrent hypertension, ischemic heart disease, and stroke exhibited the highest BAG (2.721 y) (*SI Appendix*, Fig. S9*B* and Dataset S16).

Moreover, after 4.6 y of mean follow-up, 470 (1.16%) deaths were recorded. Deceased individuals had higher BAG than survivors (1.81 vs. 0.50 y, *P* < 0.0001). Each 1-SD increment in BAG increased mortality risk by 37% (HR = 1.374, *P* = 1.63 × 10^−11^), independent of chronological age and sex. Meanwhile, BAG improved prediction accuracy (C-index, 0.708 to 0.721). Further controlling for comorbidities and socioeconomic factors, BAG remained a significant mortality predictor (HR = 1.242, *P* = 6.21 × 10^−5^), yielding best-fitting prediction (C-index, 0.751) (Fig. 4*E* and Dataset S17).

### Protein-Wide and Genome-Wide Associations of BAGs.

**Fig. 5.**
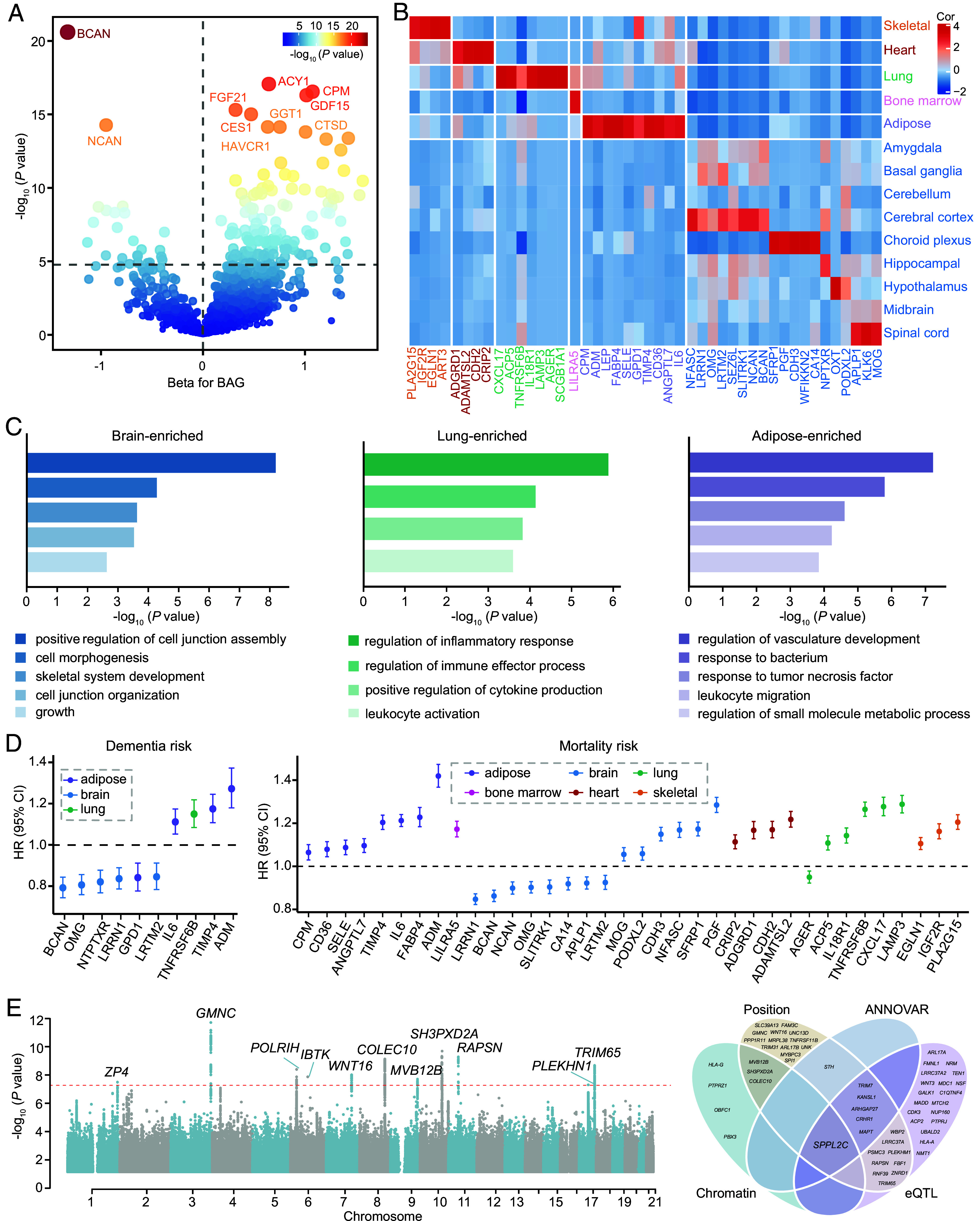
Protein-wide and genome-wide associations of BAGs. (*A*) The volcano plot shows the results of protein-wide association analysis of BAGs, with the top 10 proteins significantly associated with BAG marked. Proteins above the horizontal dotted line has Bonferroni-corrected *P* < 1.71 × 10^−5^. Chronological age, sex, and TDI are adjusted. (*B*) Tissue specificity classification of 45 BAG-related proteins according to the Human Protein Atlas database. (*C*) Gene Ontology enrichment analysis of brain-enriched, lung-enriched, and adipose-enriched proteins. Enriched pathways are defined as those with FDR correction *P* < 0.05. (*D*) Associations of BAG-related proteins with the risk of incident dementia and mortality by Cox proportional hazards regression. Potential confounders include chronological age, sex, TDI, education level, ethnicity, smoke status, alcohol frequency, SBP and BMI. Predefined 19 chronic diseases are further adjusted for the mortality risk. (*E*) The Manhattan plot shows 11 genomic loci are associated with brain age gap using a genome-wide *P* value threshold (−log_10_(*P* value) > 7.30). Venn diagram shows the results of functional annotation and gene mapping by four approaches including ANNOVAR, positional, eQTL, and chromatin interaction mapping. BAG, Brain age gap.

Protein-wide analysis identified 154 BAG-associated proteins (130 positive, 24 negative) after Bonferroni correction (adjusted *P* = 1.71 × 10^−5^) (Fig. 5*A* and Dataset S18), with tissue-specific expression patterns. Specifically, 19 brain-enriched proteins (e.g., BCAN, OMG, GFAP) associated with cell junction and morphogenesis, as well as skeletal system development; seven lung-enriched proteins (e.g., CXCL17, IL18R1) linked to inflammatory pathways; 10 adipose-enriched proteins (e.g., ADM, CD36) were involved in vasculature development, metabolic processes, and inflammation; others enriched in skeletal muscle, cardiac muscle, and bone marrow (Fig. 5 *B* and *C* and Datasets S19 and S20). Furthermore, 10 BAG-related proteins correlated with AD risk, including protective BCAN (HR = 0.792, *P* = 3.93 × 10^−13^) and risk-elevating ADM (HR = 1.272, *P* = 4.93 × 10^−10^). 36 proteins were identified as predictors of incident mortality, with LRRN1 being protective (HR = 0.847, *P* = 1.33 × 10^−28^) and ADM being detrimental (HR = 1.419, *P* = 1.99 × 10^−75^) (Fig. 5*D* and Datasets S21 and S22).

GWAS identified 11 significant loci for BAG (*P* < 5 × 10^−8^), most of which correlated with brain structure, cognition, and neurodegeneration. rs61067594 (3q28, *β* = −0.205, EAF = 0.391, *P* = 2.23×10^−12^) near *GMNC* displayed the strongest genetic signal, which was associated with neuronal proliferation and differentiation. We also identified *SPPL2C* (17q21.31) as a key gene, which was mapped with chromatin interactions in neural progenitor cells, and implicated by eQTL in cerebellar hemispheres, entire cerebellum, and frontal cortex (Fig. 5*E* and Datasets S23–S25).

### Modifiable Factors and Their Trajectories Associate with BAGs.

We identified 62 modifiable factors associated with BAGs, with WHR exhibiting the strongest effect (high vs. normal WHR: 0.853 vs. −0.089 y, *P* = 2.90 × 10^−14^), followed by hypertension (0.324 vs. −0.220 y, *P* = 1.43 × 10^−12^) (*SI Appendix*, Fig. S10*A* and Datasets S26 and S27). Subsequently, individuals were grouped by the number of abnormal top-10 risk factors: normal, low-risk (1,797, 23.8%), medium-risk (1,760, 23.3%), and high-risk (1,873, 24.8%). High-risk group with more than four abnormalities exhibited the largest BAG (0.453 y), which is 0.723 y older than normal group (*P* = 1.34 × 10^−9^) (*SI Appendix*, Fig. S10*B* and Dataset S28).

**Fig. 6.**
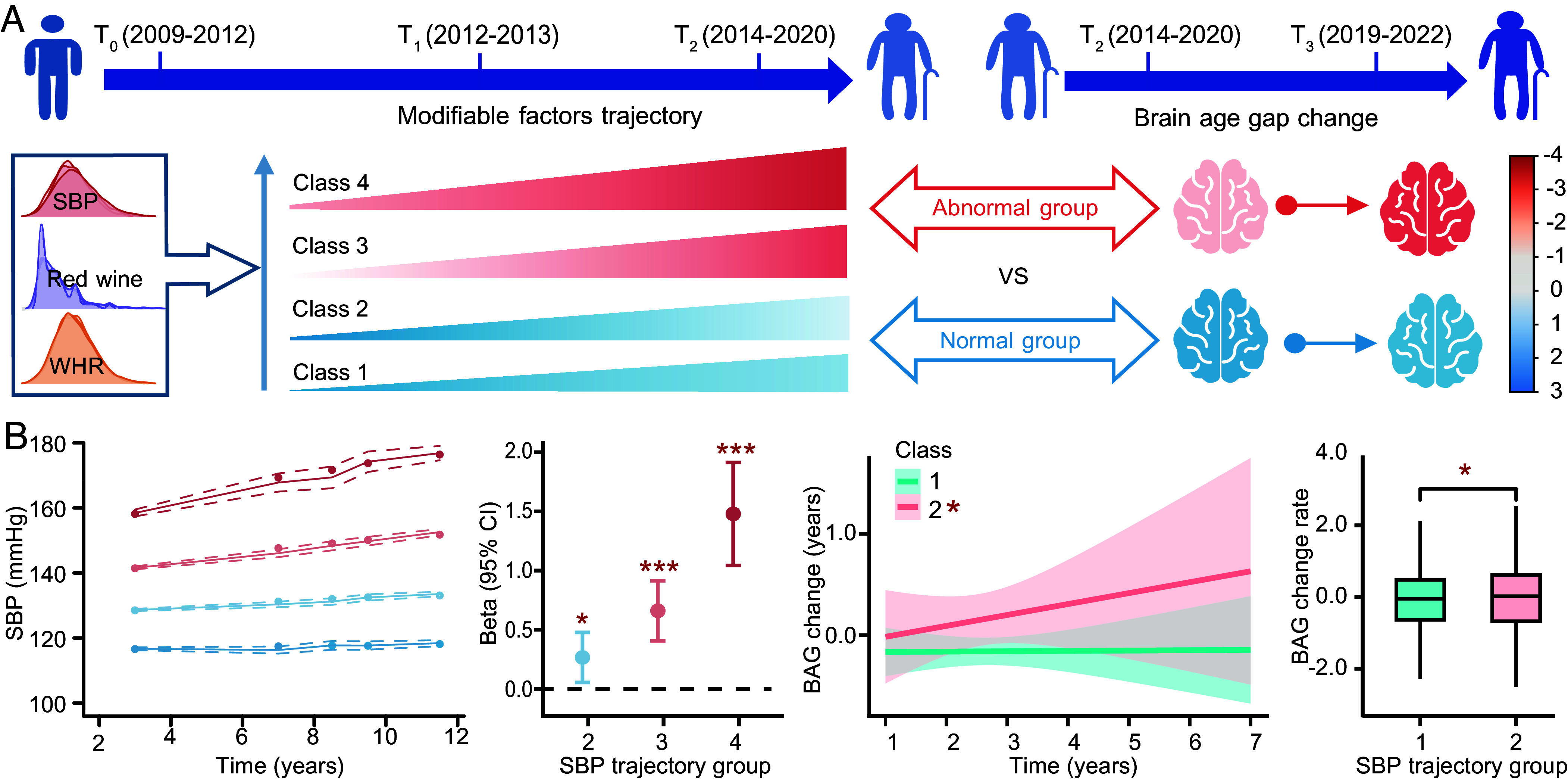
Modifiable factors and their trajectories associated with BAGs. (*A*) Modifiable factors’ trajectories are identified by latent class growth analysis (LCGA) using data from more than two surveys: T_0_ (2006–2010), T_1_ (2012–2013), and T_2_ (2014–2020). The change in BAG is estimated by longitudinal assessments from T_2_ (2014–2020) and T_3_ (2019–2022). (*B*) Line chart shows four distinct trajectories of SBP. Forest plot shows the beta and 95% CI of SBP trajectories and BAG, controlling for chronological age, sex, ethnicity, TDI, and other key risk factors. Curve plot shows the rate of change regressed against BAG change and the time interval between the baseline and follow-up assessments. The shaded area indicates the lower and upper boundaries of 95% CI. Box plot shows the significant difference in BAG change rate between two SBP trajectories by the *t* test. Significant associations are marked by asterisks (*). *P < 0.05, **P < 0.01, ***P < 0.001. BAG, Brain age gap; SBP, Systolic blood pressure.

LCGA modeled the trajectories of modifiable factors (such as SBP, WHR, frequency of drinking red wine, etc.) (Fig. 6*A* and Datasets S29 and S30). Notably, we identified four distinct SBP trajectories among 10,554 individuals. Compared to low-stable SBP (<120 mmHg), participants exhibited progressively worsening BAGs along the SBP continuum: normal-stable (120 ~ 140 mmHg, *β* = 0.266, *P* = 0.014), high-stable (140 ~ 160 mmHg, *β* = 0.660, *P* = 3.62 × 10^−7^), and high-increasing (>160 mmHg, *β* = 1.479, *P* = 2.98 × 10^−11^). Furthermore, individuals with sustained SBP exceeding 140 mmHg had a faster BAG change than those with normal SBP pattern (0.069 vs. −0.065 y, *P* = 0.032) (Fig. 6*B*). Sex-stratified analysis revealed hypertension and lifestyles drove BAGs more in females, whereas body composition had a greater impact on BAGs in males (*SI Appendix*, Figs. S11 and S12). These findings highlight that maintaining SBP within a normal range apparently keeps the brain younger.

## Discussion

Our study suggests that DTI-ALPS index could be a promising predictor of brain aging. By integrating the DTI-ALPS index into multimodal neuroimaging data, we developed a brain age model demonstrating two distinct nonlinear aging peaks with sex-specific progression rates. Notably, brain aging accelerated in females after 60 y, with musculoskeletal health being the primary factor, while males experienced a more gradual progression, driven mainly by pulmonary metrics. The model further revealed that 1-SD BAG predicted a 37% increase in mortality risk and was influenced not only by neurodegenerative disorders (e.g., dementia) but also systemic conditions such as diabetes. Critically, maintaining SBP below 120 mmHg for over a decade was associated with a 1.5-y reduction in brain aging, highlighting blood pressure management as a modifiable intervention target.

Brain aging is a complex and heterogeneous process that contributes to the decline of both mental and physical health, while also increasing the risk of age-related brain diseases, such as neurodegenerative disorders (28). Identifying biomarkers that concurrently reflect chronological age and functional brain integrity is critical for deciphering this complex phenomenon. Current biomarker discovery strategies focus on three pillars: physiological assessments, neuroimaging analyses, and humoral biomarker profiling (28). Advances in neuroimaging now enable precise quantification of age-associated structural alterations, with multimodal imaging-derived brain age frameworks emerging as robust tools for mapping brain health (29). To extend these efforts, we included the DTI-ALPS index in brain age models, largely based on prior work showing it may indirectly reflect glymphatic system function (16). Integrating this index into existing models conferred three key advantages: 1) It may serve as a sensitive marker for pathological changes in white matter microstructure to predict brain age, and might also link macrostructural brain alterations to glymphatic dysfunction, thereby enhancing mechanistic resolution; 2) It may provide a modifiable target for lifestyle or pharmacological interventions, thereby enhancing clinical actionability; 3) Developing from the large-scale UK sample and subsequent validation across additional European and Chinese cohorts ensured strong generalizability across diverse populations. Thereby, our framework advances the characterization of brain aging heterogeneity and provides a more comprehensive and actionable approach to understanding and mitigating age-related brain decline, by bridging the neuroimaging-derived structural metrics with functional clearance physiology.

Our findings demonstrate that DTI-ALPS index serves as a promising biomarker of brain aging. This is supported by three key lines of evidence: First, the DTI-ALPS index is significantly correlated with telomere length, a marker of biological aging (30), as well as age-related structural and cognitive brain changes (31). Second, this index effectively predicted cognitive decline not only in normal aging but also in neurodegenerative diseases such as AD and Parkinson’s disease (PD) (32–34), with reduced baseline ALPS values preceding amyloid pathology and clinical progression in AD (32). Third, the inclusion of ChP volume, an additional indicator related to glymphatic clearance capacity (26), further confirmed our brain age model’s predictive validity, in agreement with studies proposing enlarged ChP as a promising neuroimaging feature in AD and PD (24, 25). These converging findings help fill the knowledge gap by demonstrating that DTI-ALPS serves as a marker for brain aging, providing direct, clinically interpretable insights into early preclinical and prodromal signs of neurodegenerative disorders and cerebrovascular diseases (2, 35).

More strikingly, our study reinforced the importance of blood pressure management in brain aging by employing trajectory model to map prospectively its long-term changes. Notably, even high-normal blood pressure (120–140/80–90 mmHg) accelerates brain aging, while maintaining optimal levels (<120/80 mmHg) preserves at least half a year younger brain age. Recent studies further identified 90–114/60–74 mmHg as the most beneficial range, with each 1 mmHg increase above this threshold adding 5 to 7 d to brain aging (36). Additionally, early adulthood emerged as a critical period, where SBP over 111 mmHg strongly predicted future cognitive decline and white matter damage (37). This may be attributed to elevated blood pressure promoting cerebral arterial stiffening, which in turn compromises glymphatic function (38, 39). Collectively, our findings highlight the potential of early aggressive blood pressure control as a powerful strategy to mitigate brain aging, offering actionable insights for future clinical trials. Of note, as these results are based on group-level analyses, their clinical interpretation at the individual level requires caution. Further longitudinal or interventional studies involving multiracial population are necessary to establish population-normative and clinically relevant cut-off values for DTI-ALPS and to validate its clinical modifiability.

Brain aging evolves through nonlinear trajectories shaped by complex gene–environment–system interactions (29, 40), exhibiting fundamental sexual dimorphism in both temporal dynamics and phenotypic manifestations. Our analysis detected two critical transition periods: at 45 to 50 and 70 to 75 y of age, corresponding to established proteomic signatures of neural aging transitions (5). The latter period particularly aligns with reported acceleration of brain atrophy around 70 y (41), and increased aging-related disease risk. Importantly, we demonstrated sexually differentiated aging pathways post-60 y: females experience precipitous decline peaking at 70 y, whereas males show a more gradual, linear deterioration. This sexual divergence profoundly impacted lifespan disparities, disease vulnerability [e.g., cardiovascular disease (42) and dementia (43), and mortality profiles]. The modifiable risk architecture similarly diverges by sex: musculoskeletal traits (e.g., WHR), lifestyle habits (e.g., sun exposure and red wine consumption frequency), and psychosocial factors (e.g., social isolation) were predominant in women, while brain aging strongly correlated with body composition metrics and cardiopulmonary function (e.g., pulse rate and spirometry metrics) in men. Our findings thus emphasize the critical need for sex-stratified interventions timed to critical aging periods to effectively mitigate brain aging and associated disorders, prioritizing lifestyle or psychosocial optimization for women vs. physical structural balance for men.

Moreover, our study revealed multidimensional links between peripheral organ aging (e.g., respiratory, cardiovascular, and musculoskeletal systems) and brain aging across phenotypic, proteomic, and genetic markers, thereby extending established epidemiological associations (44). The shared biological mechanisms involved inflammatory, immune, metabolic, and vascular pathways. Additionally, skull bone marrow serves as a critical immunological interface, with aged myeloid cells infiltrating into the brain and exacerbating neuroinflammatory responses (45, 46). Furthermore, central-systemic crosstalk may utilize glymphatic and lymphatic drainage pathways, such that their failure would impair the clearance of CNS-derived antigens and pathological proteins (e.g., amyloid-β) and disrupt immune surveillance (47). This multilevel approach elucidates the complex dynamics of brain–body interactions during aging.

We incorporated the DTI-ALPS index into brain age models primarily based on a large body of previous research suggesting that it may indirectly reflect glymphatic system function (16), as well as its well-documented associations with age and a wide range of neurodegenerative diseases (48–51). However, caution should be taken when interpreting the relationship between the ALPS index and glymphatic function. First, we concede that the validation of DTI-ALPS as an index of glymphatic function remains imperfect, and it cannot assess the global glymphatic activity (20, 52). Second, we note certain methodological limitations such as the ROI placement and representation, the selection of b-value in the DTI images, and the influence of crossing fibers (19, 52). Third, DTI-ALPS is confounded by interference of multisource information such as white matter microstructure (53, 54), and a recent study suggested that reduced ALPS index is actually reflective of disruptions in white matter microstructure (18). Although these fundamental issues limit the capacity of DTI-ALPS to measure directly glymphatic function, they do not detract from its applicability for studies of brain aging and various neurodegenerative diseases. For the present, we exploit the DTI-ALPS index to measure limited aspects of glymphatic dynamics. Future multimethod, integrated research could better elucidate human glymphatic dynamics and strengthen its clinical and scientific value for brain aging studies.

Our study has certain other limitations. First, the participant cohort was restricted to middle-aged and older adults, such that inclusion of individuals across the entire lifespan would provide a more comprehensive understanding of brain aging dynamics. Second, we did not incorporate white matter microstructural metrics into our brain age model, in consideration of their strong homology with DTI-ALPS, which might not fully capture global white matter integrity. Third, we could not exclude participants with sleep apnea due to lack of polysomnography data, which may be a potential confounding factor in our analyses (22, 55). Fourth, the 2,923 plasma proteins used in our protein-wide association analysis were measured several years prior to brain phenotype assessment in the UK Biobank, potentially introducing temporal discordance that may have influenced their association with brain aging. Finally, although longitudinal data enabled estimation of brain age change rates, the relatively small sample size and short follow-up duration may have reduced our ability to detect subtle or long-term structural brain changes.

In conclusion, our findings position DTI-ALPS as a promising biomarker for nonlinear brain aging, highlighting its potential as an intervention target. The study underscores systemic contributions to brain aging, particularly through diabetes, and suggests that early aggressive blood pressure management could improve the DTI-ALPS in aging populations. These insights advocate for personalized, sex-specific strategies to ameliorate brain age-related neurological decline.

## Materials and Methods

### Study Population and Ethics Statement.

Our main dataset derived from the UK Biobank, a prospective cohort of over 500,000 adults aged 40 to 73 y, which had been approved by the UK North West Multi-Center Research Ethics Committee (MREC). We included 40,488 participants with DTI-ALPS during the third visit (2014–2020), from which a subset of 12,401 healthy adults was used for normative brain age prediction. For external validation, we included 714 participants from the ADNI study and 275 from TALENT study (2021 data). Both validation cohorts had been recruited with ethics approval and participants’ informed consent (56). Consisting of healthy individuals and those diagnosed with MCI or dementia, these two additional cohorts allowed for external validation of our normative brain aging model and its association with neurodegenerative conditions.

### Brain Age and Body Age Phenotypes.

The brain phenotypes (n = 378) (Datasets S32 and S33) derived from four neuroimaging modalities at baseline (2014-2020) and follow-up (since 2019) imaging visit, namely T1-weighted, T2-weighted, diffusion MRI (dMRI), and resting-state functional MRI (fMRI). Details of image processing are available in UK Biobank documentation (https://biobank.ctsu.ox.ac.uk/showcase/showcase/docs/brain_mri.pdf), while ADNI acquisition protocols are described elsewhere (57). Functional connectivity (FC) was derived from resting-state fMRI data using partial correlation matrices of 21 × 21 in the present analysis (58), and full details are provided in supporting information. The DTI-ALPS index was generated using the diffusion MRI sequence parameters as presented in our previous article (59). Cognitive phenotypes included 14 different cognitive performance measures covering verbal, reasoning, memory, attention, processing speed, and executive function (Dataset S34). Guided by the principle of grouping phenotypes based on their relevance to the structure and function of each organ system (29, 40), we used available metrics from the third visit (2014–2020) to measure biological age (expressed as an age gap index) for the musculoskeletal, cardiovascular, and pulmonary systems (Dataset S35).

### Development and Validation of Brain Age Models.

To create the biological age model, we first excluded individuals with self-reported or documented lifetime chronic medical conditions (Dataset S36). We employed XGBoost models to estimate an individual’s chronological age in a healthy population using 10-fold cross-validation. Model accuracy was assessed with Pearson correlation (*r*) and mean absolute error (MAE) between predicted and chronological ages. The age gap, which is the difference between chronological and predicted age, serves as organ-specific clocks of biological age. A positive gap suggests accelerated aging, while a negative gap indicates slower aging compared to healthy peers. In our analysis, we corrected for regression-to-the-mean bias by regressing chronological age from all estimated age gaps (29, 60). Multivariable linear regression model was used to investigate the relationships between peripheral organ age gaps and BAGs. To validate generalizability of our brain age model in older age cohorts (29), we adopted a dual-validation approach: we separately retrained the model using 1) pooled healthy participants from UKB and ADNI, and 2) UKB and TALENT combined datasets. Prior to model training, all data underwent harmonization and scaling to minimize scanner- and dataset-related variability in brain phenotypic measurements. To assess the potential confounding influence of vascular risk factors, we performed a sensitivity analysis that incorporated multiple vascular risk factors into brain age estimation. These factors included smoking status, alcohol use, frequency of alcohol consumption, physical activity, insomnia, sleep duration, snoring, diet, SBP, diastolic blood pressure (DBP), body mass index (BMI), and WHR. To further estimate the validity of the GS in brain age estimation, we incorporated ChP volume as an alternative to DTI-ALPS in the UKB-based modeling framework. To mitigate microstructural biases, we corrected the ALPS index in a sample of 652 individuals with known Aβ status from the ADNI dataset, following the methodology of a prior study (53).

### Health-Related Outcomes and Mortality Risk Prediction.

The health-related outcomes were defined as the first occurrences in the UK Biobank, obtained from hospital inpatient records, primary care data, death register data, and self-reported medical conditions from the UK National Health Services (NHS). Individuals with lifetime diagnoses among 19 chronic diseases were grouped into one of eight clinical systemic disease categories. The diagnostic codes for each disease category are listed in Datasets S36 and S37. In the ADNI cohort, patients with AD and MCI were diagnosed according to the National Institute of Neurological and Communicative Disorders and Stroke and Alzheimer’s Disease and Related Disorders Association (NINCDS-ADRDA) criteria. For the TALENT study, MCI diagnosis was based on comprehensive neuropsychological testing and confirmed through neurologist consensus (61). For comparison, we used the previously established normative models for healthy individuals to estimate biological brain age for each disease category. The significance and effect sizes of BAGs differences between each disease category and the healthy group were assessed using the *t* test and Cohen’s d with Bonferroni correction for multiple comparisons.

Mortality status and dates was accessed from UK National Death Registries (https://biobank.ctsu.ox.ac.uk/crystal/refer.cgi?id=115559) and linked to the UK Biobank records. Survival duration was calculated from baseline brain MRI assessment date to death date, or right-censored on 14 September 2022. The difference of BAGs between deceased and living individuals was compared using a two-sample *t* test, and mortality risk of BAGs was assessed by Cox proportional hazards regression.

### Protein-Wide and Genome-Wide Analysis of BAGs.

The UK Biobank Pharma Proteomics Project quantified 2,923 plasma proteins (Olink) among over 50,000 participants (62). We analyzed 5,302 participants with both proteomic and neuroimaging data (Dataset S38), identifying BAG-associated proteins via age-, sex-, and DTI-adjusted linear regression (Bonferroni-corrected). Gene expression data from various tissues were sourced from the Human Protein Atlas database (https://www.proteinatlas.org/about/download). Gene ontology (GO) enrichment analysis identified significant pathways (FDR-adjusted *P* < 0.05) through the Metascape web tool (63), with all human genes as the background. Additionally, Cox proportional hazards model assessed BAG-associated dementia or mortality risk.

We conducted quality control on imputed genotypes from white British of European ancestry using PLINK as described previously (64, 65). After excluding related individuals (kinship coefficient ≥ 0.0884), we conducted the GWAS of BAG in 31,612 individuals with 14,611,868 variants on GRCh37, adjusting for age, sex, age–sex interaction, age-squared, age-squared-sex interaction, total intracranial volume, array batch, and top 10 ancestry principal components (Dataset S39). Genome-wide significant SNPs (*P* < 5 × 10^−8^, linkage disequilibrium r^2^ < 0.6) were identified, and candidate variants were annotated with r^2^ ≥ 0.6, GWAS *P* value < 1 × 10^−5^, and MAF >0.001 in FUMA (https://fuma.ctglab.nl/) (66). Four approaches were employed for functional annotation and gene mapping, including ANNOVAR (67), positional mapping, eQTL mapping (68), and chromatin interaction mapping (69). Candidate SNPs were subsequently searched in NHGRI-EBI GWAS catalog to identify their previously identified associations with any other traits (70).

### Modifiable Factors and Their Trajectory with BAG Change.

We conducted sex-stratified multiple linear regression to explore the relationship between BAGs and 157 modifiable factors across seven domains (Dataset S31). The top 10 influential factors were categorized into normal and abnormal groups for BAG comparison. Individuals with no, one, two to three, and more than four abnormal conditions were stratified as reference, low-, medium-, and high-risk groups, respectively. Then, latent class growth analysis (LCGA) was used to delineate 12-y trajectories of modifiable factors among 12,401 participants with more than two measurements across three examination periods (2006–2010, 2012–2013, 2014–2020). The optimal number of trajectories was selected based on the lowest Bayesian information criterion (BIC), while ensuring that the posterior probabilities for each class were above 0.70 and that each class contained at least 2% of the population (71). The associations of these modifiable factors and their trajectories with BAGs were assessed using a linear regression model. A normative brain age model was trained on baseline (T_2_) data to predict chronological age at follow-up (T_3_). BAG change was determined by the difference between baseline and follow-up estimated BAGs among 1,364 individuals. The rate of change was modeled against the time interval, spanning up to 7 y, with the slope of the regression line indicating the rate of change per year.

## Supplementary Material

Appendix 01 (PDF)

Dataset S01 (XLSX)

Dataset S02 (XLSX)

Dataset S03 (XLSX)

Dataset S04 (XLSX)

Dataset S05 (XLSX)

Dataset S06 (XLSX)

Dataset S07 (XLSX)

Dataset S08 (XLSX)

Dataset S09 (XLSX)

Dataset S10 (XLSX)

Dataset S11 (XLSX)

Dataset S12 (XLSX)

Dataset S13 (XLSX)

Dataset S14 (XLSX)

Dataset S15 (XLSX)

Dataset S16 (XLSX)

Dataset S17 (XLSX)

Dataset S18 (XLSX)

Dataset S19 (XLSX)

Dataset S20 (XLSX)

Dataset S21 (XLSX)

Dataset S22 (XLSX)

Dataset S23 (XLSX)

Dataset S24 (XLSX)

Dataset S25 (XLSX)

Dataset S26 (XLSX)

Dataset S27 (XLSX)

Dataset S28 (XLSX)

Dataset S29 (XLSX)

Dataset S30 (XLSX)

Dataset S31 (XLSX)

Dataset S32 (XLSX)

Dataset S33 (XLSX)

Dataset S34 (XLSX)

Dataset S35 (XLSX)

Dataset S36 (XLSX)

Dataset S37 (XLSX)

Dataset S38 (XLSX)

Dataset S39 (XLSX)

## Data Availability

Data for this study were sourced from publicly available datasets, including UK Biobank (https://www.ukbiobank.ac.uk/) (72) and ADNI (https://adni.loni.usc.edu/) (73). Additionally, we utilized an in-house TALENT dataset (ChiCTR1900027225), which is accessible via the following link: https://www.chictr.org.cn/ (74). No accession codes are needed to access these data, and the authors do not hold any special access privileges to the data from these databases beyond what is available to other researchers. All data are included in the manuscript and/or supporting information. Python software is available from https://github.com/Python (75), and R software is available from https://www.r-project.org/ (76). The code used for data processing and core analysis has been made publicly available at https://github.com/Lwxixixi/Brain-Aging.git (77).
