# Supplementary material for: Unveiling the glymphatic system’s role in brain aging: A comprehensive biomarker and modifiable intervention target
Source: Proc Natl Acad Sci U S A. 2026 Apr 27;123(18):e2516601123. doi: 10.1073/pnas.2516601123 (PMC13142974; doi:10.1073/pnas.2516601123)
Supplement: Supplementary file 1 — Appendix 01 (PDF) [file pnas.2516601123.sapp.pdf]

Supporting Information for

Unveiling the Glymphatic System's Role in Brain Aging: A Comprehensive Biomarker and Modifiable Intervention Target

Yuanyuan Fang [a,b,1], Wenxi Luo [c,1], Hao Huang [a,b,1], Lusen Ran [a,b], Yuqin He [a,b], Chang Cheng [a,b], Yao Yao [a,b], Yuxiang Hou [d], Haibo Zheng [d], Dengji Pan [a,b], Shabei Xu [a,b], Xiang Luo [a,b], Tingting Qin [e], Xingjie Hao [f], Feng Lu [d,2], Wei Wang [a,b,2], Minghuan Wang [a,b,2]

Correspondence to be addressed to Feng Lu, Wei Wang, or Minghuan Wang

Email: lufeng@hust.edu.cn, wwang@vip.126.com, or mhwang@tjh.tjmu.edu.cn.

This PDF file includes:

Supporting text
Figs. S1 to S12
Methods
Statistical Analysis Plan (SAP)
SI References

Supporting Information Text

Results

Supplementary Figures

Fig. S1. Flowchart of study design and sample selection.

Fig. S2. Flowchart of the brain age model development and validation.

Fig. S3. SHAP values of the top 15 predictors sorted by their feature importance to the DTI-ALPS prediction.

Fig. S4. Brain age prediction across male and female populations.

Fig. S5. Sensitivity analysis of brain age prediction.

Fig. S6. Comparison of the original ALPS and cALPS for brain age prediction in validation datasets (ADNI and UKB cohorts).

Fig. S7. Influencing factors of DTI-ALPS index across the total and sex-stratified populations.

Fig. S8. Organ-specific age prediction across the total and sex-stratified populations.

Fig. S9. Brain age in combination of multiple chronic diseases.

Fig. S10. Associations of modifiable factors with BAGs in total populations.

Fig. S11. Associations of modifiable factors with BAGs across male and female populations.

Fig. S12. Associations of modifiable factors' trajectories with BAGs in the overall population and stratified by sex.

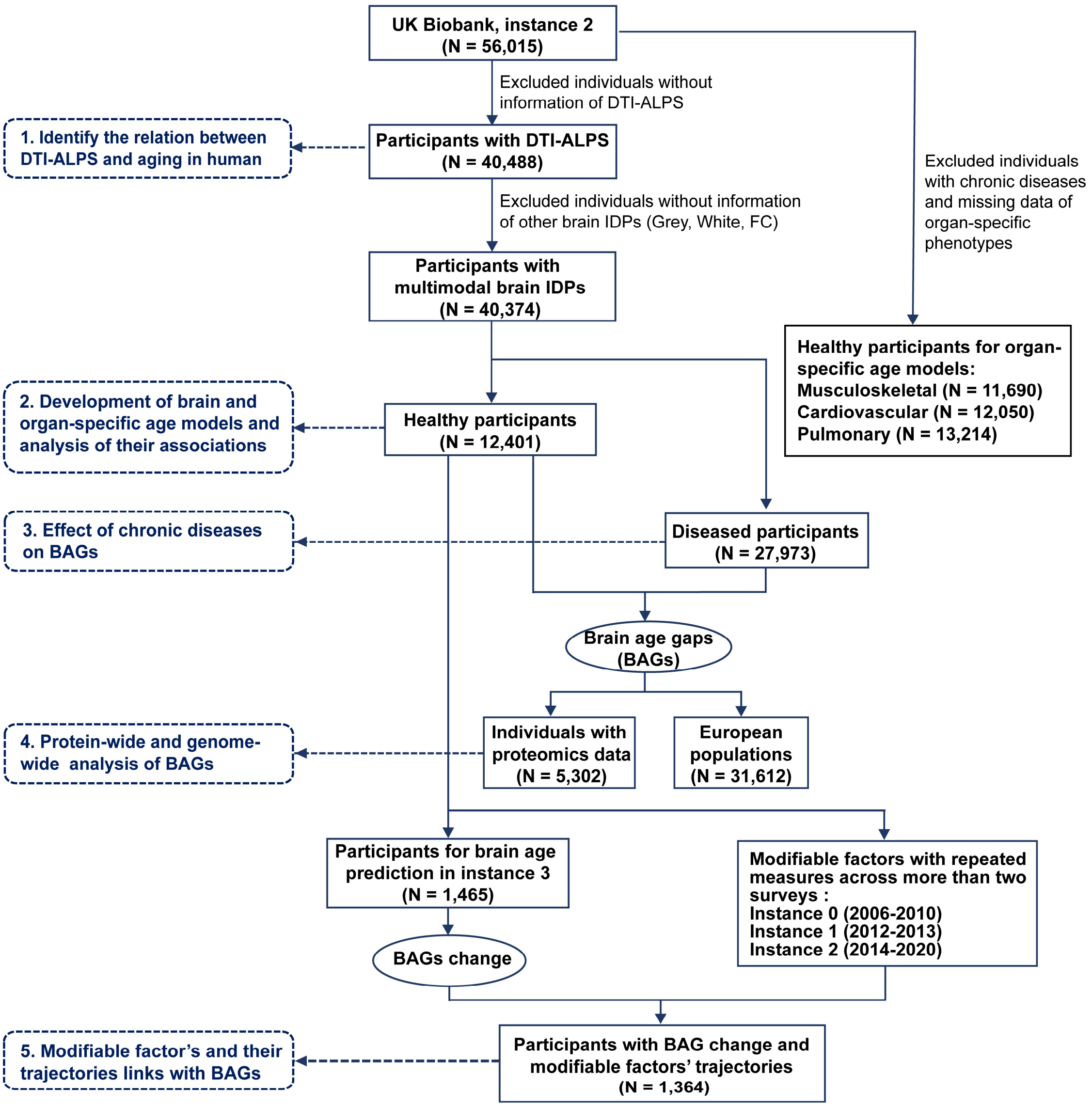

Fig. S1. Flowchart of study design and sample selection. DTI-ALPS, Diffusion tensor imaging along perivascular spaces; FC, Functional connection; IDPs, Imaging-derived phenotypes; BAGs, Brain age gaps.

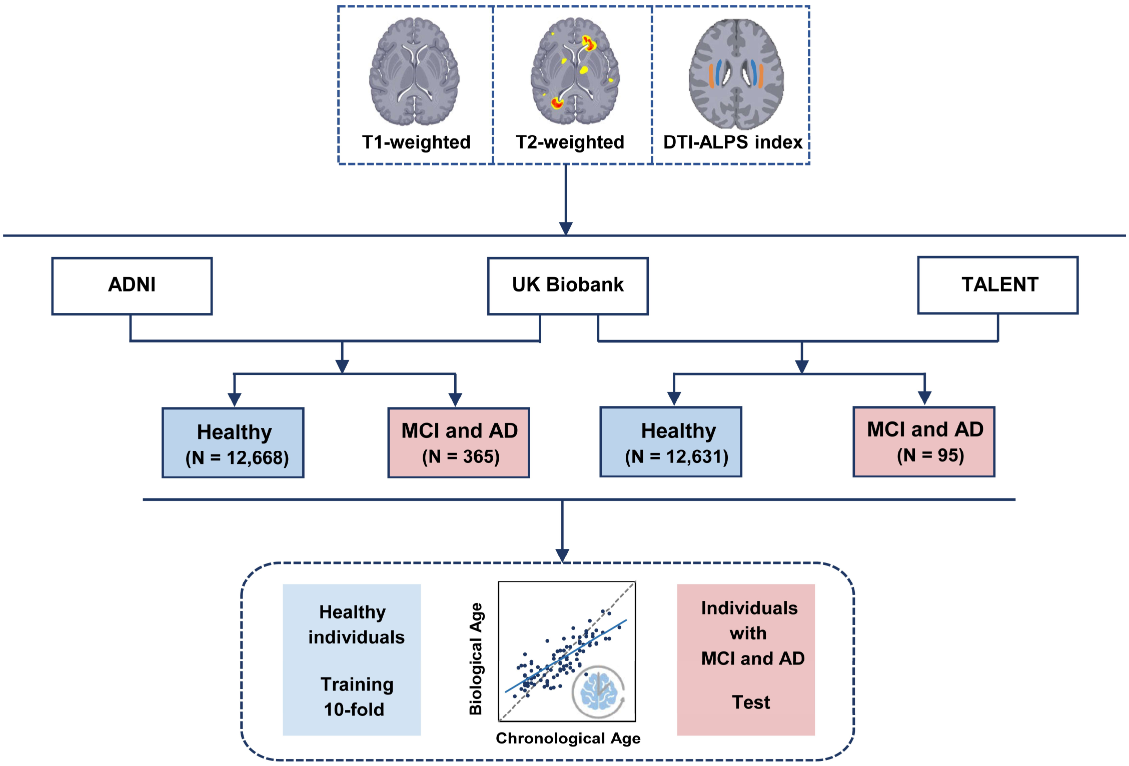

Fig. S2. Flowchart of the brain age model development and validation. DTI-ALPS, Diffusion tensor imaging along perivascular spaces; ADNI, Alzheimer's Disease NeuroImaging Initiative; TALENT, Tongji cerebrAl smalL vEssel disease and agiNg cohorT; MCI, Mild cognitive impairment; AD, Alzheimer's disease.

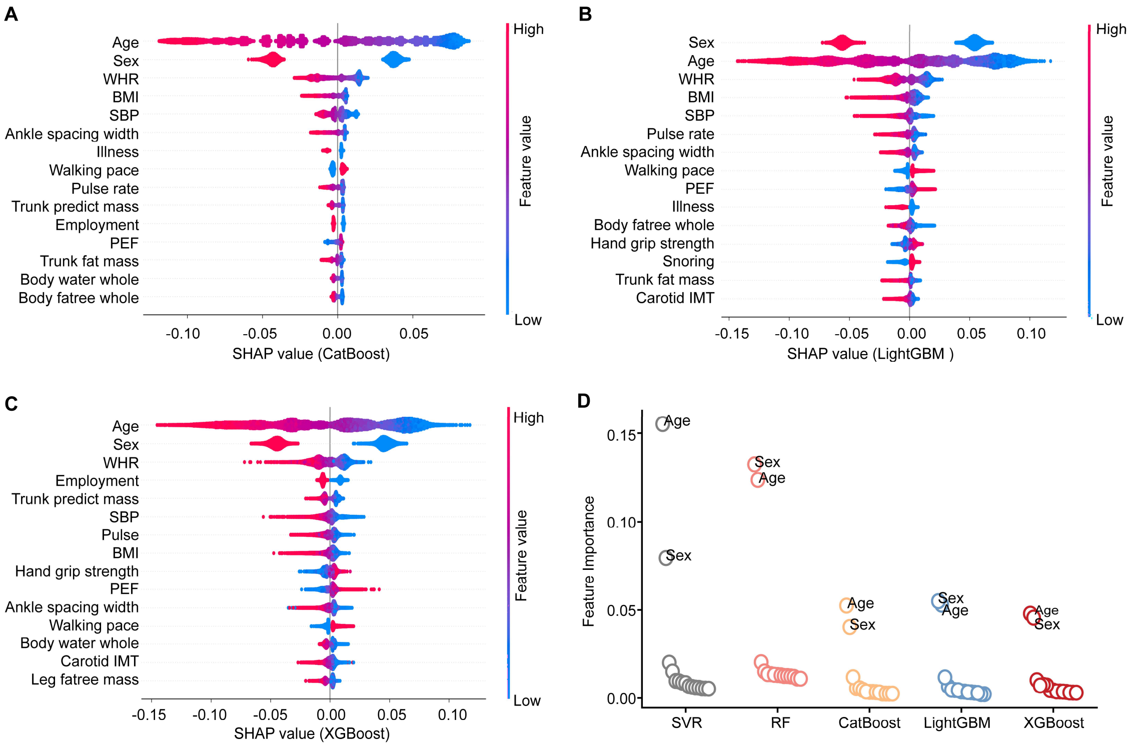

Fig. S3. SHAP values of the top 15 predictors sorted by their feature importance to the DTI-ALPS prediction. (*A*) SHAP values in CatBoost model. (*B*) SHAP values in LightGBM model. (*C*) SHAP values in XGBoost model. Here are two aspects to interpret SHAP plots. Each participant is presented as a data point and coded with gradient colors representing the magnitude of the predictor. On the one hand, the horizontal range represent the overall predictive power of each predictor. In specific, age seemed to have the widest range, demonstrating it had the most considerable prediction power and can significantly impact the model's output, followed by sex. On the other hand, the value magnitude and tendency direction on the x-axis represents the specific effect extent of each predictor. Taking age as an example, older participants (colored in red) had negative impact on DTI-ALPS (left side) compared with younger ones (colored in blue). (*D*) The ranking of the top 15 predictors in five distinct machine learning algorithms, with the top two predictors age and sex marked. DTI-ALPS, Diffusion tensor imaging along perivascular spaces; CatBoost, Categorical Boosting; LightGBM, Light Gradient Boosting Machine; XGBoost, eXtreme Gradient Boosting; SVR, Support Vector Regression; RF, Random Forest; SHAP, SHapley Additive exPlanations; SBP, Systolic blood pressure; WHR, Waist-to-Hip Ratio; BMI, Body mass index; PEF, Peak expiratory flow; IMT, Intima-Media thickness.

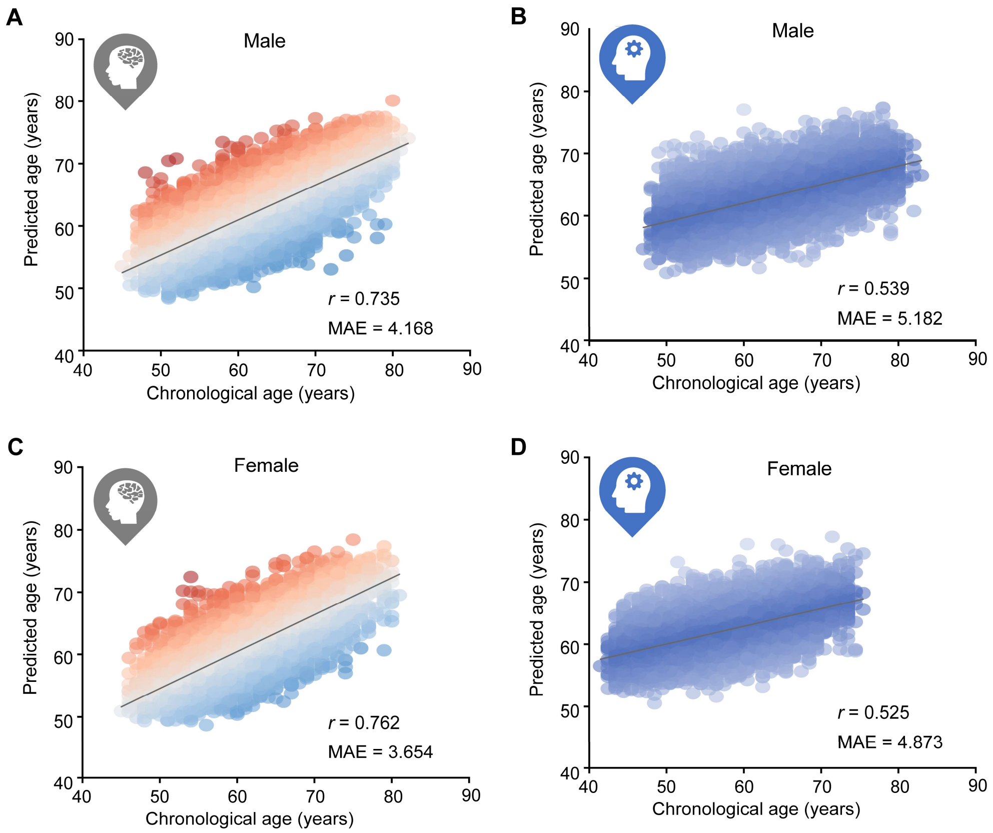

Fig. S4. Brain age prediction across male and female populations. (*A*) and (*C*) Prediction of brain age using multimodal brain IDPs incorporating DTI-ALPS in males and females. (*B*) and (*D*) Prediction of cognition age using cognitive phenotypes in males and females. Lines of best fit are indicated with solid black lines. *r*, Pearson correlation coefficient; MAE, Mean absolute error.

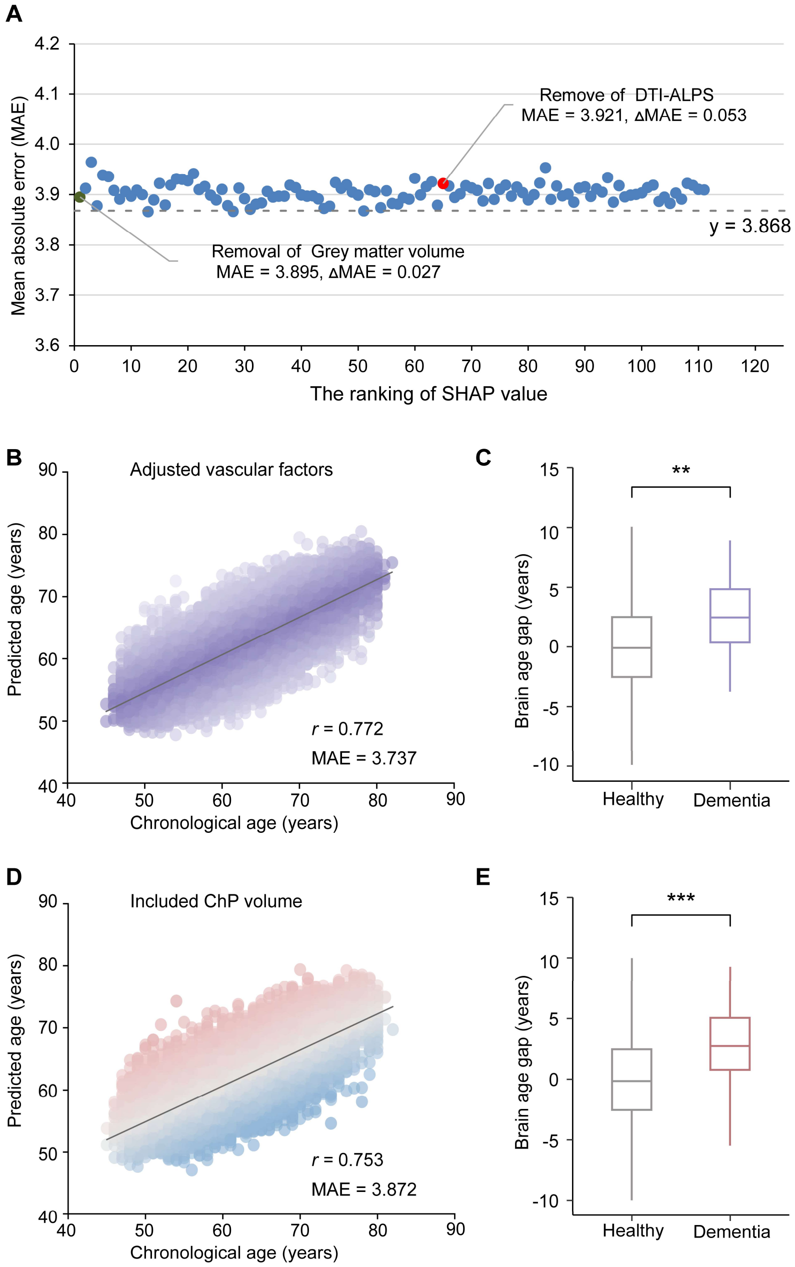

Fig. S5. Sensitivity analysis of brain age prediction. (*A*) Impact of sequential removal of brain imaging-derived phenotypes on MAE of the brain age model. Features are removed in order of their importance (ranked 1 to 111). The red dot denotes the DTI-ALPS index, and the forest-green dot represents grey matter volume. The y-axis shows the MAE after removing each corresponding feature. (*B, D*) Scatter plots of chronological age versus predicted brain age after adjusting for vascular factors (B) or after replacing the DTI-ALPS index with choroid plexus (ChP) volume (D). Lines of best fit are indicated with solid black lines. *r*, Pearson correlation coefficient; MAE, Mean absolute error. (*C, E*) Box plots comparing the brain age gap between healthy controls and individuals with dementia following adjustment for vascular factors (C) or inclusion of ChP volume

(E). Significant associations determined by *t*-test are marked by asterisks (** $P < 0.01$, *** $P < 0.001$).

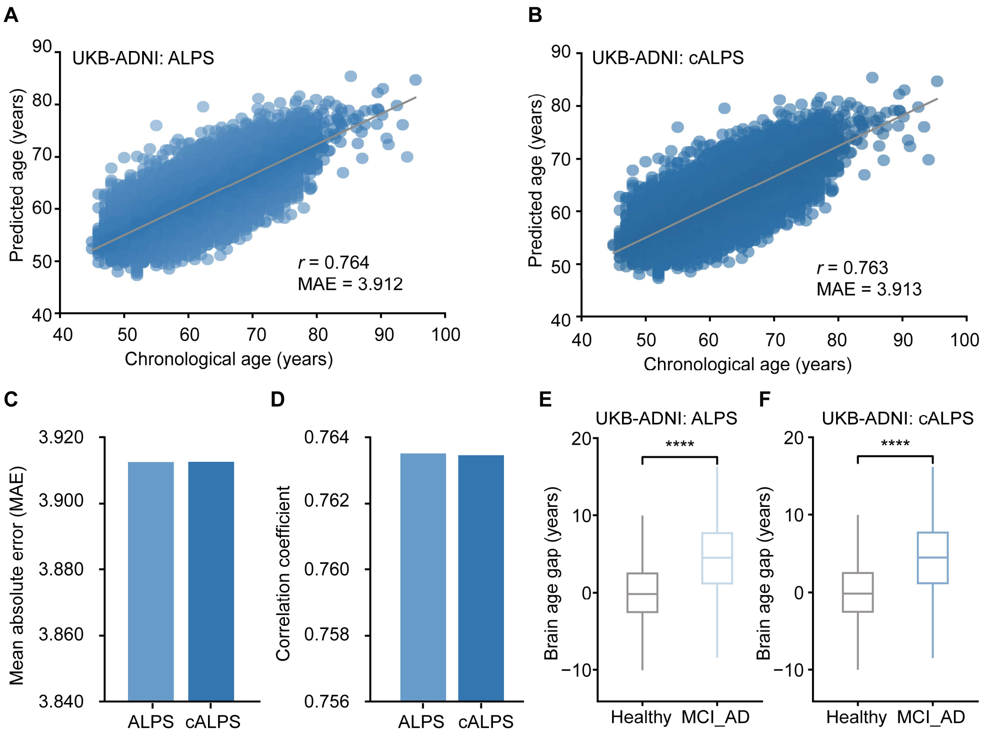

Fig. S6. Comparison of the original ALPS and cALPS for brain age prediction in validation datasets (ADNI and UKB cohorts). (*A, B*) Brain age models trained using the original ALPS and cALPS features, respectively. Scatter plots display the Pearson correlation (*r*) and MAE between predicted brain age and chronological age. (*C, D*) MAE and *r* values of brain age models built with ALPS versus cALPS features. (*E, F*) Differences in brain age gaps between healthy controls and individuals with MCI or AD. Significant differences determined by *t*-tests are marked with asterisks ($P < 0.0001$). Abbreviations: *r*, Pearson correlation coefficient; MAE, Mean absolute error; MCI, Mild cognitive impairment; AD, Alzheimer's disease.

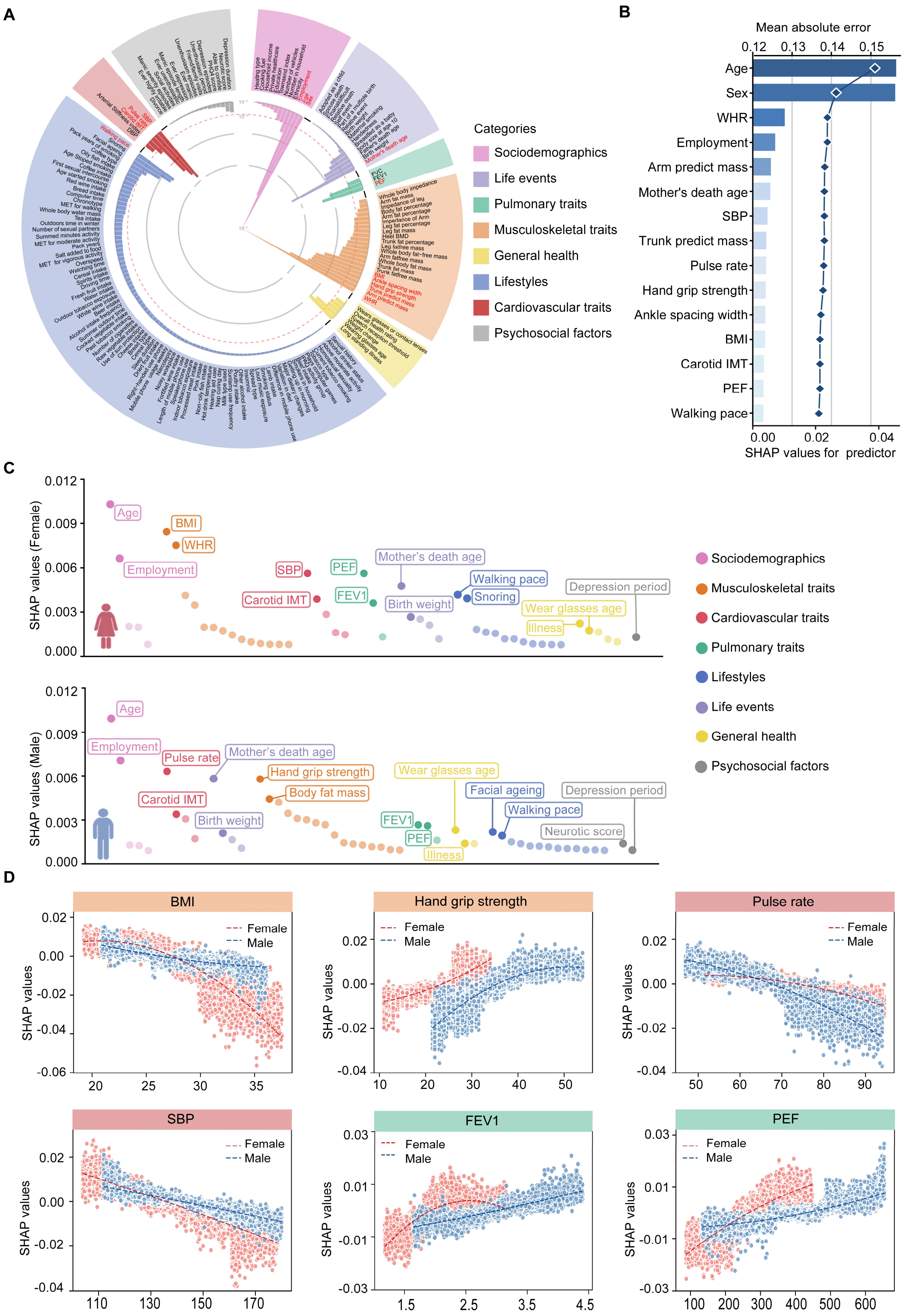
A
Categories
Sociodemographics
Life events
Pulmonary traits
Musculoskeletal traits
General health
Lifestyles
Cardiovascular traits
Psychosocial factors
B
Mean absolute error
0.12
0.13
0.14
0.15
Age
Sex
WHR
Employment
Arm predict mass
Mother's death age
SBP
Trunk predict mass
Pulse rate
Hand grip strength
Ankle spacing width
BMI
Carotid IMT
PEF
Walking pace
0.00
0.02
0.04
SHAP values for predictor
C
SHAP values (Female)
0.012
0.009
0.006
0.003
0.000
Age
BMI
WHR
Employment
SBP
PEF
Mother's death age
Walking pace
Depression period
Carotid IMT
FEV1
Birth weight
Snoring
Wear glasses age
Illness
Sociodemographics
Musculoskeletal traits
Cardiovascular traits
Pulmonary traits
Lifestyles
Life events
General health
Psychosocial factors
SHAP values (Male)
Age
Employment
Pulse rate
Mother's death age
Hand grip strength
Wear glasses age
Body fat mass
Facial ageing
Depression period
Carotid IMT
Walking pace
Birth weight
FEV1
Neurotic score
PEF
Illness
D
BMI
Hand grip strength
Pulse rate
SBP
FEV1
PEF
SHAP values
Female
Male

Fig. S7. Influencing factors of DTI-ALPS index across the total and sex-stratified populations. (*A*) The feature importance of 161 candidate predictors is classified into eight clusters in the total population. (*B*) The top 15 predictors of DTI-ALPS index sorted by SHAP values and the mean absolute error of the model are shown. (*C*) SHAP values of the top 50 features to DTI-ALPS prediction task across female and male populations. (*D*) The relationship of original and SHAP values of musculoskeletal traits (BMI and hand grip strength), cardiovascular characteristics (pulse rate and SBP), and pulmonary function (FEV1 and PEF) in female and male individuals. Lines of best fit are indicated with dotted red (female) and blue (male) lines. WHR, Waist-to-Hip Ratio; SBP, Systolic blood pressure; BMI, Body mass index; IMT, Intima-Media thickness; PEF, Peak expiratory flow; FEV1, Forced expiratory volume in 1 second.

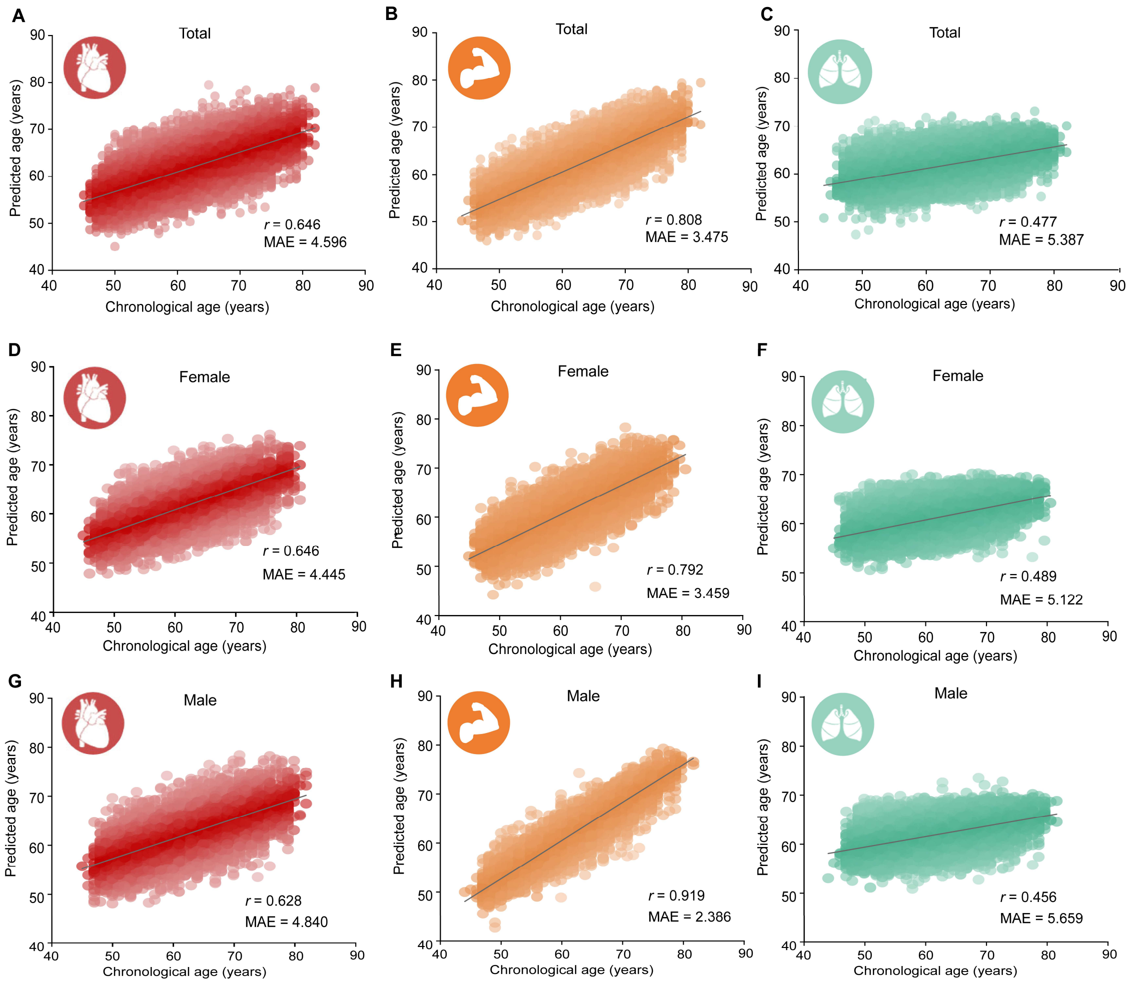

Fig. S8. Organ-specific age prediction across the total and sex-stratified populations. Scatter plots show the correlation between predicted and chronological age of cardiovascular, musculoskeletal, and pulmonary age for total population (*A-C*), females (*D-F*) and males (*G-I*), respectively. Lines of best fit are indicated with solid black lines. *r*, Pearson correlation coefficient; MAE, Mean absolute error.

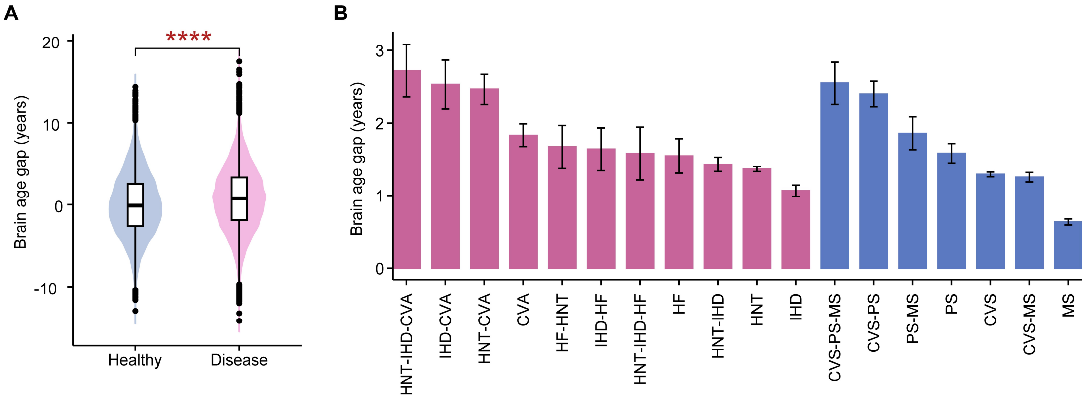

Fig. S9. Brain age in combination of multiple chronic diseases. (*A*) The differences in brain age gap between healthy and disease population. Significant associations determined by *t*-test are marked by asterisks ($P < 0.0001$). (*B*) Accumulation of brain age gaps related to comorbidities involving all three system disorders and cardiovascular system disorders. Error bars indicate standard errors (s.e.). CVS, Cardiovascular system; PS, Pulmonary system; MS, Musculoskeletal system; HTN, Hypertension; IHD, Ischemic heart disease; CVA, Cerebral vascular attack; HF, Heart failure.

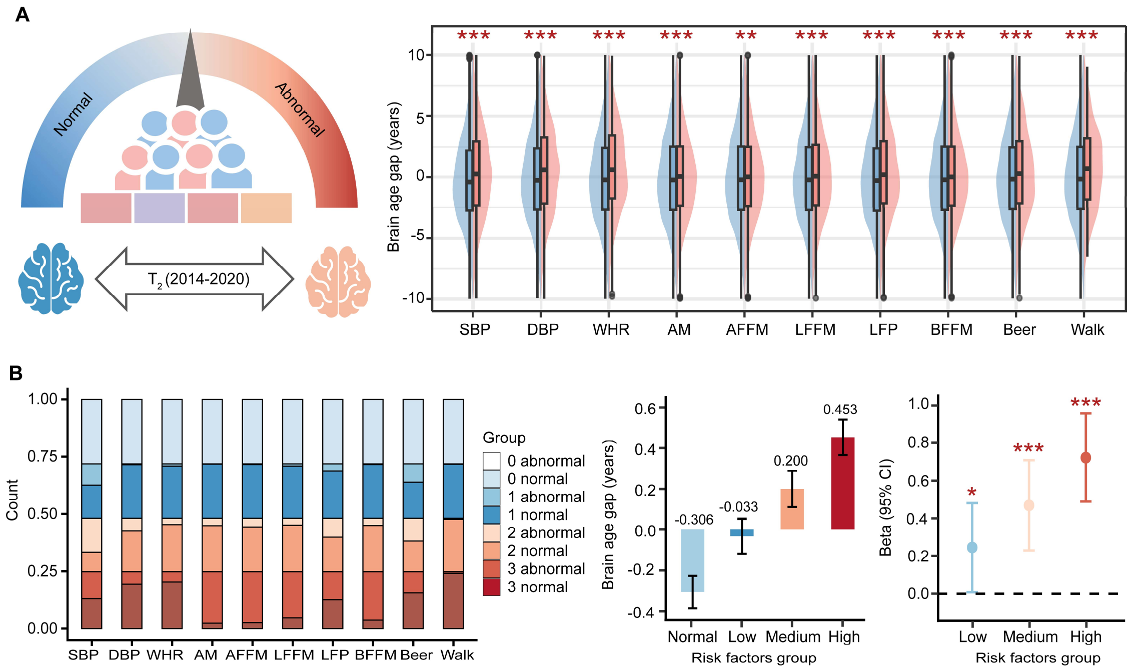

Fig. S10. Associations of modifiable factors with BAGs in total populations. (*A*) Modifiable factors of BAG are categorized into normal and abnormal groups. Violin plots show the significant difference in BAG between the normal (blue) and abnormal (red) groups for ten factors using *t*-test, with horizontal line representing the mean BAG values. (*B*) Stacked bar chart (left) shows the percentage of normal and abnormal conditions for ten modifiable factors across four risk groups: normal group (no abnormal condition), low-risk group (one abnormal condition), medium-risk group (2-3 abnormal conditions), and high-risk group (more than four abnormal conditions). Bar plot (middle) shows the mean BAGs of four risk factor groups, with values listed. Error bars indicate standard errors (s.e.). Forest plot (right) shows the beta and 95% CI of BAGs in abnormal condition groups compared to normal group. Confounders include chronological age and sex. Significant associations are marked by asterisks (*). * $P < 0.05$, ** $P < 0.01$, *** $P < 0.001$. SBP, Systolic blood pressure; DBP, Diastolic blood pressure; WHR, Waist-to-Hip Ratio; AM, Arm predict mass; AFFM, Arm fat-free mass; LFFM, Leg fat-free mass; BFFM, Body fat-free mass; LFP, Leg fat percentage.

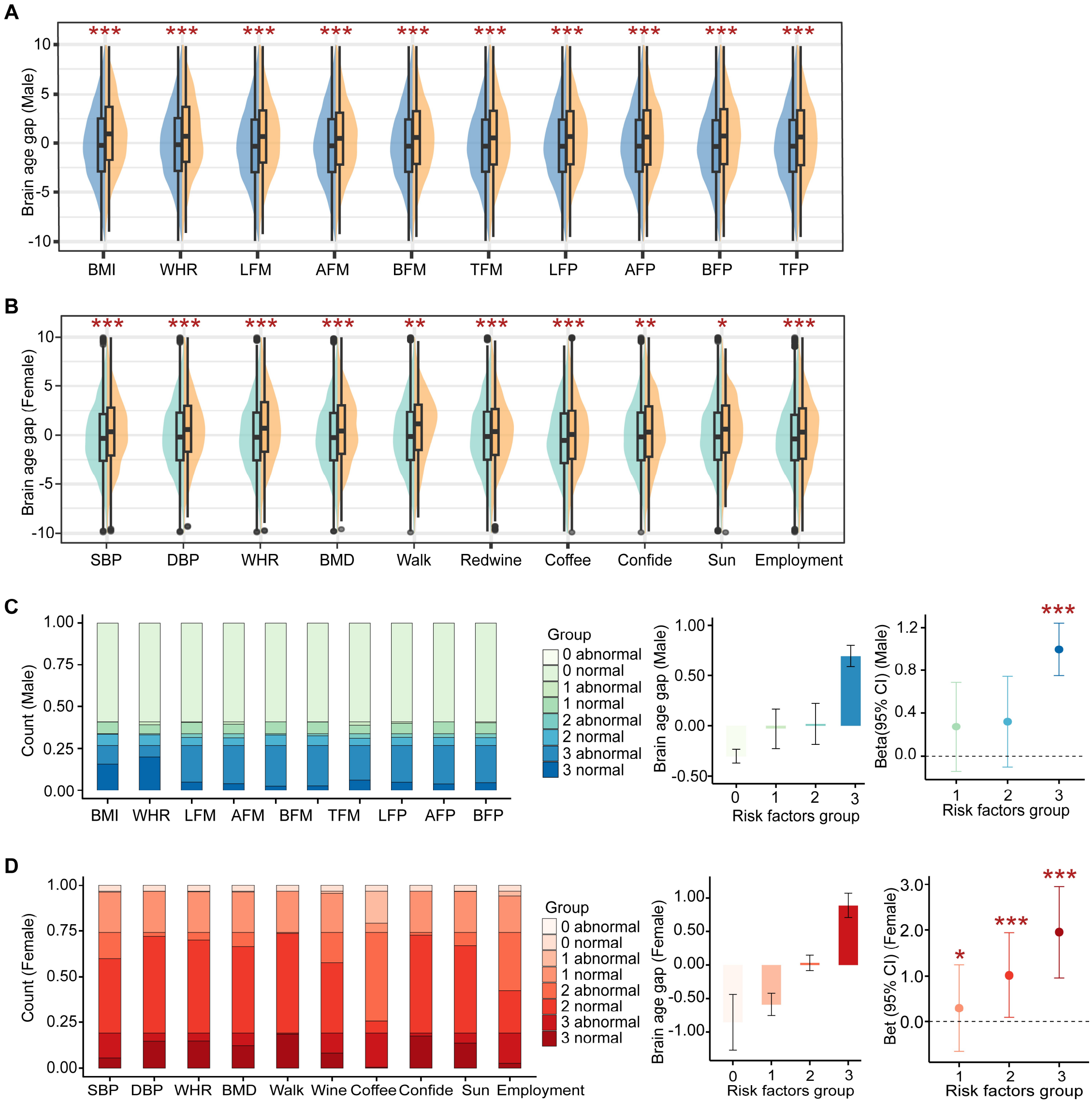

Fig. S11. Associations of modifiable factors with BAGs across male and female populations. (*A*) Violin plots show the significant difference in BAG between the normal and abnormal groups for ten factors using t-test in men, with horizontal line representing the mean BAG values. (*B*) same as (A), but in women. (*C*) Stacked bar chart (left) shows the percentage of normal and abnormal conditions for ten modifiable factors across four risk groups in men: normal group, low-risk group, medium-risk group, and high-risk group. Bar plot (middle) shows the mean BAGs of four risk factor groups in men. Error bars indicate standard errors (s.e.). Forest plot (right) shows the beta and 95% CI of BAGs in abnormal condition groups compared to normal group in men. Confounders include chronological age and sex. (*D*) same as (C), but in women. Significant associations are marked by asterisks (*). * $P < 0.05$, ** $P < 0.01$, *** $P < 0.001$. LFM, Leg fat mass; AFM, Arm fat mass; BFM, Body fat mass; TFM, Trunk fat mass; LFP, Leg fat percentage; AFP, Arm fat percentage; BFP, Body fat percentage; TFP, Trunk fat percentage; BMD, Bone mineral density.

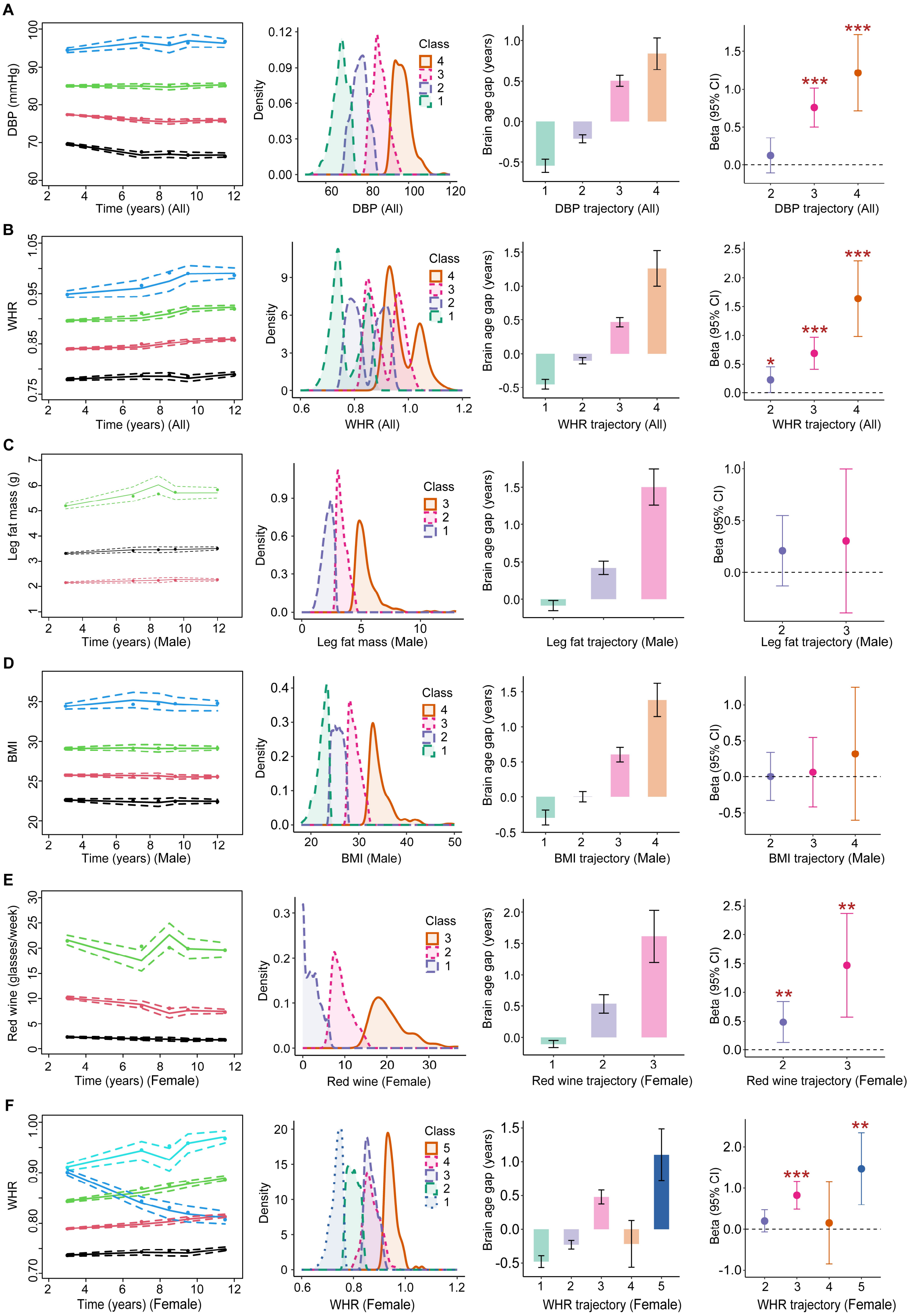
A
DBP (mmHg)
Time (years) (All)
Density
DBP (All)
Class
4
3
2
1
Brain age gap (years)
DBP trajectory (All)
Beta (95% CI)
***
***
DBP trajectory (All)
B
WHR
Time (years) (All)
Density
WHR (All)
Class
4
3
2
1
Brain age gap (years)
WHR trajectory (All)
Beta (95% CI)
*
***
***
WHR trajectory (All)
C
Leg fat mass (g)
Time (years) (Male)
Density
Leg fat mass (Male)
Class
3
2
1
Brain age gap (years)
Leg fat trajectory (Male)
Beta (95% CI)
Leg fat trajectory (Male)
D
BMI
Time (years) (Male)
Density
BMI (Male)
Class
4
3
2
1
Brain age gap (years)
BMI trajectory (Male)
Beta (95% CI)
BMI trajectory (Male)
E
Red wine (glasses/week)
Time (years) (Female)
Density
Red wine (Female)
Class
3
2
1
Brain age gap (years)
Red wine trajectory (Female)
Beta (95% CI)
**
**
Red wine trajectory (Female)
F
WHR
Time (years) (Female)
Density
WHR (Female)
Class
5
4
3
2
1
Brain age gap (years)
WHR trajectory (Female)
Beta (95% CI)
***
**
WHR trajectory (Female)

Fig. S12. Associations of modifiable factors' trajectories with BAGs in the overall population and stratified by sex. (*A*) Line chart shows four distinct trajectories of DBP in overall population. Density plot illustrates the distribution of DBP across four trajectory groups. Bar plot displays the mean BAGs of four DBP trajectory groups. Error bars indicate standard errors (s.e.). Forest plot presents the beta and 95% CI of DBP trajectory groups and BAG, controlling for chronological age, sex, ethnicity, TDI, and other key risk factors. (*B*) WHR trajectories and their association with BAG in overall population. (*C*) Leg fat mass trajectories and their association with BAG among the male population. (*D*) BMI trajectories and their association with BAG among the male population. (*E*) Frequency of red wine intake trajectories and their association with BAG among the female population. (*F*) WHR trajectories and their association with BAG among the female population. Significant associations are marked by asterisks (*). * $P < 0.05$, ** $P < 0.01$, *** $P < 0.001$.

Methods

UK Biobank

Our main dataset derived from the UK Biobank, a population-based cohort, which recruited over 500,000 community-dwelling adults aged 40-73 years at baseline (2006-2010) across 22 assessment centers. Multimodal brain imaging was performed during the third assessment visit (2014-2020) among 56,015 participants at dedicated imaging centers located in Manchester, Reading, and Newcastle. Follow-up brain imaging has been ongoing in 4,622 individuals since 2019, providing a longitudinal sample enabling estimation of the rate of change in brain age. The study received approval from the UK North West Multi-Centre Research Ethics Committee (MREC) (http://www.ukbiobank.ac.uk/ethics/), and all participants provided written informed consent. The present analysis was conducted under project application number 90311.

DTI-ALPS prediction model and predictors identification

To identify specific predictors of DTI-ALPS, we selected multidimensional and clinically relevant variables from UK Biobank imaging visit to establish a prediction model using five distinct machine learning algorithms (1): eXtreme Gradient Boosting (XGBoost), Light Gradient Boosting Machine (LightGBM), Categorical Boosting (CatBoost), Support Vector Regression (SVR), and Randomforest by the Python software version v.3.8.3. Following a systematic comparison of the five algorithms in terms of predictive accuracy (quantified by MAE), generalizability, and interpretability, XGBoost emerged as the optimal choice (1, 2). It demonstrated superior performance on the independent test set, achieving the lowest MAE (0.1356 years) and the highest explained variance ($R^2 = 0.3098$) (Dataset S4). To handle missing data, we excluded variables with more than 40% missing responses. For stable variables (e.g., ethnicity), missing values were filled according to repeated measurements. Variables with less than 5% missingness were imputed using mean or mode values, while those with exceeding 5% missingness employed iterative imputation (continuous) or multinomial logistic regression (categorical). To minimize multicollinearity, redundant or multi-measurement variables were consolidated by averaging their values. The detailed demographic information of all candidate predictors for this study is listed in Dataset S31.

To quantify feature importance across varying learning paradigms, and enable a robust comparison of their contributions to predictive performance, we employed SHAP values to interpret the feature significance in the XGBoost, LightGBM, and CatBoost models (2-4). The feature importance in Randomforest was determined by the mean decrease impurity (MDI) metric, averaging Gini impurity across splits (5). The feature importance in SVR was assessed by the absolute magnitude of the weight vector, with larger weight indicating stronger feature impact (6).

Development of organ-specific models

We utilized multi-omics data from the third UK Biobank assessment (2014-2020), including physical, physiological, and imaging measures, to index the function, structure or general health of the cardiovascular, musculoskeletal, and pulmonary systems. After excluding participants with missing organ-specific phenotypes or a history of chronic medical conditions, we constructed predictive models of chronological age using the XGBoost algorithm, incorporating all phenotypes within each organ-specific group. The cardiovascular age model (N = 12,050) incorporated 34 features from blood pressure, carotid ultrasound, arterial stiffness, 12-lead electrocardiograms and cardiac MRI. The musculoskeletal age model (N = 11,690) entailed 55 phenotypes derived from anthropometry, bone-densitometry, as well as bone size, mineral, and density through DXA assessment. The pulmonary age model (N = 13,214) encompassed three lung function metrics obtained through spirometry. All organ-specific phenotypes are detailed in Dataset S35, and the performance characteristics for organ-specific models are presented in Dataset S11.

Essential methodological steps for functional connectivity (FC) calculation

The present study employed subject-specific network matrices derived from an image processing pipeline established and maintained by the UK Biobank (7). Detailed descriptions of the MRI acquisition protocols and processing methods are available in a prior review (8) and in comprehensive online documentation (https://biobank.ctsu.ox.ac.uk/crystal/crystal/docs/brain_mri.pdf). During scanning, participants were asked to fixate on a crosshair, remain still, and stay relaxed without focusing on anything in particular.

The raw fMRI data underwent preprocessing involving motion correction, grand-mean intensity normalization, high-pass temporal filtering, unwarping of echo-planar images, gradient distortion correction, and removal of structured artifacts via ICA-based X-noiseifier from the FMRIB Software Library. Group-level independent component analysis (ICA) was conducted using FSL on 4,100 datasets at two dimensionalities (100 and 25). Artifactual components identified and excluded by UK Biobank experts yielded two sets of neuronally-relevant components numbering 55 and 21 items, respectively. These group-level spatial maps were then projected onto each participant's fMRI time series to extract one representative time series per ICA component per subject. These time series served as the basis for estimating individual network matrices.

In this framework, each "node" corresponds to the spatial map of an ICA component, which may encompass multiple spatially distributed brain regions. Network modeling was performed using the FSLNets toolbox, computing both full and partial temporal correlations between all pairs of nodal time series. Pearson correlation coefficients were converted to z-statistics. For the current study, the 21 × 21 partial correlation matrices were used.

Statistical Analysis Plan (SAP)

Study Title: Unveiling the Glymphatic System's Role in Brain Aging: A Comprehensive Biomarker and Modifiable Intervention Target

1. Study Objectives

This study aims to examine the role of DTI-ALPS in brain aging and its links to peripheral organ aging, chronic diseases, mortality, and modifiable factors. The primary objectives are to develop normative brain age models using healthy adults' multimodal neuroimaging data, evaluate BAGs in clinical and deceased participants, investigate BAGs association with cognitive function, organ-specific aging, plasma proteins, genetic variants, and mortality risk, and assess how longitudinal modifiable factor trajectories relate to BAGs.

2. Analysis Populations

(1) UK Biobank (UKB): 40,488 participants included for DTI-ALPS modeling; 12,401 healthy participants without major medical conditions used to train the normative brain age model.
(2) External Validation cohorts: 714 participants from Alzheimer's Disease Neuroimaging Initiative (ADNI); 275 participants from TALENT study.
(3) Proteomics: 5302 participants with both plasma protein and neuroimaging data from UKB.
(4) GWAS: 31,612 European ancestry participants for genome-wide analysis of BAGs from UKB.
(5) Longitudinal Analyses: 1364 participants with repeated assessments across three study phases (2006-2010, 2012-2013, 2014-2020) from UKB.

3. Descriptive Analysis

Descriptive analyses were performed for demographics of included individuals. Continuous variables were summarized as mean ± standard deviation (SD), and categorical variables were summarized as counts and percentages. Comparisons across sex and age groups were conducted using independent *t*-tests or chi-square tests as appropriate.

4. Glymphatic System and Brain Age Analysis

4.1 DTI-ALPS Associations

Associations between the DTI-ALPS index and 163 influencing factors (sociodemographic, lifestyle, life event, psychosocial factors, general health, and physical measurement) were assessed using five distinct machine learning algorithms: eXtreme Gradient Boosting (XGBoost), Light Gradient Boosting Machine (LightGBM), Categorical Boosting (CatBoost), Support Vector Regression (SVR), and Randomforest by the Python software version v.3.8.3. Feature importance of each influencing factors across varying learning paradigms was quantified using SHapley Additive exPlanations (SHAP) values, mean decrease impurity (MDI) metric, and absolute

magnitude of the weight vector where appropriate, enabling a comparison of their contributions to predictive performance.

Associations between the DTI-ALPS index and aging hallmarks were evaluated using linear regression models. These hallmarks included chronological age, leukocyte telomere length, brain imaging-derived phenotypes (IDPs), and cognitive performance across 14 domains. Each model included the DTI-ALPS index and was adjusted for age, sex, and relevant socioeconomic variables where applicable. Results were reported as standardized beta coefficients with corresponding standard errors and two-sided p-values.

4.2 Brain Age Prediction Model

Brain age prediction models were developed using the XGBoost algorithm, trained exclusively in healthy participants (N = 12,401) to predict chronological age based on multimodal neuroimaging phenotypes. Model training employed 10-fold cross-validation, with predicted ages generated for the held-out test folds in each iteration. Model performance was evaluated using Pearson correlation coefficients (r) and mean absolute error (MAE) between predicted and chronological age. Feature importance of individual neuroimaging phenotypes was quantified using SHAP values; these values were used to assess predictive contributions, but were not interpreted as causal effects.

The brain age gap (BAG) was defined as the difference between predicted and chronological age, with regression-to-the-mean correction applied to minimize bias.

Sensitivity analyses included models incorporating vascular risk factors, and models replacing the DTI-ALPS index with choroid plexus (ChP) volume or the corrected ALPS (cALPS) as the predictor. External validation of the final model was conducted in independent cohorts from ADNI and TALENT, applying the trained model to estimate brain age and BAGs in these populations.

4.3 Organ-Specific Age Models

Organ-specific age models were developed for the musculoskeletal, cardiovascular, and pulmonary systems using relevant phenotypic measures. The XGBoost algorithm was applied with 10-fold cross-validation within the healthy reference population to predict chronological age for each organ system.

Associations between organ-specific age gaps and BAGs were assessed using multivariable linear regression models. All models were adjusted for chronological age, sex, and relevant socioeconomic variables. Sex-stratified analyses were conducted when appropriate to examine potential sex-specific effects. Model outputs were reported as standardized beta coefficients with corresponding standard errors and two-sided p-values.

5. Chronic Diseases and Mortality

To examine associations of BAGs with chronic diseases, we used the previously established normative brain age models for healthy individuals to estimate biological brain age, generating BAGs for each disease category. The differences in BAGs between individuals with specific diagnoses and healthy controls were evaluated using independent samples *t*-tests. Effect sizes were quantified using Cohen's d. Bonferroni correction was applied to account for multiple comparisons, with significance thresholds set at $P < 0.05/8$ for systemic disease categories and $P < 0.05/18$ for individual disease categories.

Mortality analyses were conducted using Cox proportional hazards regression models to estimate hazard ratios (HRs) per one standard deviation increase in standardized BAGs (mean = 0, SD = 1). Four sequential models were constructed:

Model 1: Adjusted for age and sex.

Model 2: Model 1 plus adjustment for 19 chronic diseases.

Model 3: Model 2 plus adjustment for ethnicity and Townsend Deprivation Index (TDI).

Model 4: Model 3 plus additional adjustment for general health (presence of long-standing illness) and key covariates, including walking pace, systolic blood pressure (SBP), pulse rate, carotid intima-media thickness, waist-to-hip ratio (WHR), body fat distribution (total, truncal, and leg fat mass), and lean mass (arm).

Model performance was evaluated using the concordance index (C-index). All analyses were two-sided, and standardized BAGs were used to facilitate comparability of hazard ratio estimates across models.

6. Protein-Wide and Genome-Wide Analyses

6.1 Proteomic Analysis

To identify molecular correlates of brain aging, associations between plasma protein levels and BAGs were evaluated using linear regression models. Each protein was analyzed separately as the predictor, with BAG as the outcome, and models were adjusted for age, sex. Multiple testing was controlled using Bonferroni correction, with a significance threshold set at $P < 0.05/2923$.

Tissue-specific annotations for significant proteins were obtained from the Human Protein Atlas, and pathway enrichment analyses were performed using Metascape, with a false discovery rate (FDR) threshold of < 0.05.

The associations of BAG-related proteins with incident dementia and all-cause mortality were evaluated using Cox proportional hazards regression. Three sequential models were constructed:

Model 1: Adjusted for age, sex, Townsend Deprivation Index (TDI), education, and ethnicity.

Model 2: Model 1 plus adjustment for smoking status, alcohol consumption frequency, systolic blood pressure (SBP), and body mass index (BMI).

Model 3: Model 2 plus adjustment for 19 chronic diseases (applied for mortality analyses only).

All BAG-related proteins were standardized (mean = 0, SD = 1) prior to analysis, and hazard ratios with 95% confidence intervals were reported.

6.2 GWAS Analysis

Genome-wide association analyses (GWAS) were conducted to identify genetic variants associated with BAGs. Genotype quality control and imputation followed standard PLINK procedures. Related individuals with kinship ≥ 0.0884 were excluded. Models were adjusted for age, age^2, sex, age-by-sex interactions, total intracranial volume (ICV), genotyping batch, and the top ten ancestry principal components to account for population stratification.

Genome-wide significant loci were defined using a threshold of $P < 5\times10^{-8}$, with linkage disequilibrium pruning applied at $r^2 < 0.6$ to identify independent signals. Functional annotation of significant loci was performed using ANNOVAR, including positional mapping, expression quantitative trait loci (eQTL) analyses, and chromatin interaction data. Identified loci were cross-referenced with the NHGRI-EBI GWAS catalog to determine previously reported associations.

7. Modifiable Factors and Longitudinal Trajectories

A total of 157 modifiable factors spanning seven domains, including lifestyle, anthropometry, blood pressure, physical activity, diet, and other relevant measures, were analyzed to investigate their associations with BAGs. Initial associations were assessed using multivariable linear regression models, with BAG as the outcome and sex included as a covariate to enable sex-stratified analyses.

The top ten factors demonstrating the strongest associations with BAGs were dichotomized into normal versus abnormal categories using predefined cut-offs. Participants were subsequently stratified into four risk groups based on the number of abnormal findings: reference (0 abnormal), low-risk (1 abnormal), medium-risk (2-3 abnormalities), and high-risk (≥4 abnormalities).

Longitudinal trajectories of the top three modifiable factors were modeled using latent class growth analysis (LCGA) over approximately 12 years. Model selection was guided by the lowest Bayesian information criterion (BIC), with additional requirements of posterior probabilities > 0.70 for each class and class sizes ≥ 2% of the total population.

Associations between trajectory group membership and BAGs were evaluated using multivariable linear regression. Changes in BAG over time were quantified as the slope derived from the difference between follow-up and baseline BAG measures, allowing assessment of the impact of distinct longitudinal patterns of modifiable factors on brain aging.

8. Statistical Considerations and Software

All statistical analyses were conducted using Python (v3.8.3) for machine learning modeling and data preprocessing, R (v4.5.1) for conventional statistical analyses, LCGA, and data visualization,

and STATA (v17) for data cleaning. All hypothesis tests were two-sided, with a significance threshold of α = 0.05 unless otherwise specified. Multiple comparisons were controlled using either Bonferroni correction or false discovery rate (FDR) procedures, as appropriate. Continuous variables were assessed for normality, with transformations applied when necessary to meet model assumptions. Missing values for variables with <5% missingness were imputed using the mean (for continuous variables) or mode (for categorical variables). For variables with >5% missingness, iterative imputation was used for continuous variables, and multinomial logistic regression imputation for categorical variables.

To ensure transparency and reproducibility, all data processing and analysis scripts have been made publicly available at https://github.com/Lwxixixi/Brain-Aging. Data access for this study was obtained through the UK Biobank (Application No. 90311) and via registration procedures and data use agreements for the ADNI and TALENT studies.

This analytical framework preserves a clear separation between descriptive inference, predictive modeling, and downstream epidemiological analyses. Machine learning outputs were used exclusively for prediction and feature evaluation, while all downstream analyses of BAGs were performed using conventional statistical approaches to ensure interpretability. Proteomic, genomic, and longitudinal analyses were treated as hypothesis-generating, with results interpreted in the context of potential biological mechanisms and intervention targets.

Reference

1. M. A. Beheshti I Fau - Ganaie *et al.*, Predicting Brain Age Using Machine Learning Algorithms: A Comprehensive Evaluation. *IEEE J Biomed Health Inform* **26**, 1432-1440 (2022).
2. M. Wiens, A. H. Verone-Boyle, N., J. Podichetty, J. Burton, A Tutorial and Use Case Example of the eXtreme Gradient Boosting (XGBoost) Artificial Intelligence Algorithm for Drug Development Applications. *Clin Transl Sci* **18**, e70172 (2025).
3. Guolin Ke *et al.*, LightGBM: a highly efficient gradient boosting decision tree. *Advances in neural information processing systems* **30**, 3149–3157 (2017).
4. A. Valsaraj *et al.*, Development and validation of echocardiography-based machine-learning models to predict mortality. *EBioMedicine* **90**, 104479 (2023).
5. H. A.-O. Zhang *et al.*, Tree-based ensemble machine learning models in the prediction of acute respiratory distress syndrome following cardiac surgery: a multicenter cohort study. *J Transl Med* **22**, 772 (2024).
6. Y. E. Tian *et al.*, Heterogeneous aging across multiple organ systems and prediction of chronic disease and mortality. *Nature Medicine* **29**, 1221-1231 (2023).

7. F. Alfaro-Almagro *et al.*, Image processing and Quality Control for the first 10,000 brain imaging datasets from UK Biobank. *Neuroimage* **166** (2018).
8. K. L. Miller *et al.*, Multimodal population brain imaging in the UK Biobank prospective epidemiological study. *Nat Neurosci* **19**, 1523-1536 (2016).
